# Sociodemographic disparities, healthcare system trust, and social support in mental health treatment among U.S. adults with depressive or anxiety symptoms

**DOI:** 10.1016/j.xjmad.2026.100166

**Published:** 2026-01-13

**Authors:** Anudeepa K. Ramachandiran, Faith Gunning, Mauricio Santillana, Matthew A. Baum, Pilar F. Verhaak, James N. Druckman, Katherine Ognyanova, David Lazer, Roy H. Perlis

**Affiliations:** a Department of Psychiatry, Massachusetts General Hospital, 185 Cambridge Street, Boston, MA 02114, United States; b Department of Psychiatry, Harvard Medical School, 25 Shattuck Street, Boston, MA 02115, United States; c Department of Psychiatry, Weill Cornell Medicine, 525 E 68th St, New York, NY 10065, United States; d Network Science Institute, Northeastern University, 177 Huntington Ave, Boston, MA 02115, United States; e Institute for Quantitative Social Science, Harvard University, 1737 Cambridge Street, Cambridge, MA 02138, United States; f John F. Kennedy School of Government and Department of Government, 79 John F. Kennedy Street, Cambridge, MA 02138, United States; g Department of Political Science, University of Rochester, 333 Hutchinson Road, Rochester, NY 14627, United States; h Department of Communication, School of Communication and Information, Rutgers University, 4 Huntington Street, New Brunswick, NJ 08901, United States

**Keywords:** Mental health disparities, Depression, Anxiety, Treatment access, Healthcare trust, Social support, Sociodemographic factors

## Abstract

Large and persistent sociodemographic disparities in rates of mental health treatment in the United States have been reported, but whether these differences reflect institutional mistrust or limited social support remains unclear. This study described current treatment use among American adults with moderate-to-severe depressive or anxiety symptoms and examined whether trust in health care institutions and availability of emotional support were associated with lack of treatment. A cross-sectional analysis was conducted using data from a nationally distributed, web-based opinion survey of 9733 American adults with moderate-to-severe depressive or anxiety symptoms (Patient Health Questionnaire-9 score ≥10 and/or Generalized Anxiety Disorder-2 score ≥3). The survey was fielded April 10th–28th, 2025, using quota sampling for age, gender, race, ethnicity, education, U.S. census region, and urbanicity; post-stratification weights approximated the U.S. adult population. The primary outcome was no current mental health treatment (neither antidepressant nor psychotherapy use). Weighted logistic regression estimated odds ratios for treatment absence by sociodemographic characteristics, trust in physicians and hospitals, scientists and researchers, the Centers for Disease Control and Prevention, pharmaceutical companies, and emotional support. Among 9733 adults with elevated symptoms, 66.3 % reported no current treatment. Racial and ethnic minority groups, men, and those born outside the United States had higher odds of being untreated, while public insurance predicted lower odds. Lower trust in doctors and hospitals, lower trust in science, and lack of emotional support each independently predicted treatment absence, but inclusion of these variables did not meaningfully attenuate sociodemographic disparities.

## Highlights

• Examined recent mental health treatment patterns among U.S. adults with moderate-to-severe depressive or anxiety symptoms.
• Nearly two-thirds of adults with elevated symptoms were not receiving any formal mental health care.
• Racial, ethnic, and gender disparities in mental health treatment use were pronounced.
• Lower trust in doctors and scientists and lack of emotional support were independently associated with being untreated.
• Accounting for healthcare trust and emotional support did not meaningfully reduce sociodemographic treatment disparities. .

## 1 Introduction

Antidepressant medications and psychotherapy are well-established as first-line treatments for depressive and anxiety disorders [1], [2], [3] because of their extensive evidence of efficacy across a wide range of clinical populations [4], [5], [6]. However, these treatments fail to reach a substantial portion of U.S. adults who might benefit from their use. In fact, a national survey indicated that among individuals with moderate-to-severe depressive symptoms, only about half reported receiving any formal mental health care [7].

Persistent treatment gaps are especially pronounced in many marginalized populations [8]. Large national studies have consistently documented disparities across racial, ethnic, and other socioeconomic groups. For example, among U.S. adults with any self-reported mental illness in the past year, 62.1 % of Black, 60.4 % of Hispanic, and 63.9 % of Asian Americans report receiving no mental health treatment, compared to 43.9 % of White adults [9]. Socioeconomic disadvantage has also been shown to impact use patterns, with lower income and lack of insurance associated with reduced likelihood of initiating treatment [10].

The present study leverages a large, nationally distributed U.S. survey administered between April 10th–28th, 2025 and examines adults with moderate-to-severe depressive or anxiety symptoms (N = 9733) to better characterize treatment gaps among those most likely to benefit from care. We also assess whether distinct facets of healthcare system trust, including trust in physicians and hospitals, scientists and researchers, the CDC, and pharmaceutical companies, as well as the availability of emotional support, predict treatment absence and/or reduce observed sociodemographic disparities.

We hypothesized that lower trust in health care institutions and lack of emotional support would be associated with higher odds of being untreated and would account for a portion of the observed sociodemographic disparities in treatment use, as medical mistrust is a well-documented barrier to engaging with health services [11], [12], [13], [14], [15]. Social support has also been linked to treatment seeking, with supportive networks encouraging the initiation of care [16], [17], with some studies suggesting these patterns may differ among racial and ethnic minority groups [18], [19]. We expected that trust and social support would attenuate sociodemographic disparities in treatment.

## 2 Methods

### 2.1 Study design

Data were drawn from a nationally distributed online survey administered between April 10th– 28th, 2025 using Qualtrics, with recruitment managed by Pure Spectrum, a commercial panel aggregator. The survey was a broad, multi-topic opinion poll and was not specifically designed or presented as a mental health survey. Participants were drawn from multiple online panels and compensated with incentives such as cash, gift cards, loyalty points, online credits, or sweepstakes entries. A nonprobability sampling strategy [20] with quotas for age, gender,= race, ethnicity, education, U.S. census region, and urbanicity was used to approximate the demographic composition of the U.S. adult population. Eligible respondents were 18 years or older and resided in the United States. All respondents provided electronic informed consent prior to beginning the survey. The study was reviewed by the appropriate Institutional Review Board and deemed exempt. Survey reporting followed the American Association for Public Opinion Research (AAPOR) guidelines.

### 2.2 Measures

Current antidepressant use was assessed with the item: “Have you ever used a prescription antidepressant medication like Prozac, Zoloft, or Lexapro?” Respondents who answered “Yes, currently taking” were classified as current users. Psychotherapy use was measured with the parallel item: “Have you ever received psychotherapy (talk therapy)?” Current use was defined as “Yes, currently receiving”. Binary indicators were created for no current treatment (neither antidepressant nor psychotherapy use), no antidepressant use, or no psychotherapy use. Respondents who selected “Prefer not to answer” were excluded from analyses of that outcome.

Depressive symptoms were measured using the nine-item Patient Health Questionnaire (PHQ-9), and anxiety symptoms using the two-item Generalized Anxiety Disorder scale (GAD-2). Respondents were included in the analytic sample if they scored PHQ-9 ≥ 10 and/or GAD-2 ≥ 3, clinical thresholds to indicate moderate-to-severe depressive and anxiety symptoms, respectively [21], [22], [23], [24].

To measure trust in healthcare institutions, respondents were asked: “How much do you trust the following people and organizations to do what is right?” Institutions included were doctors and hospitals, pharmaceutical companies, the US Centers for Disease Control and Prevention (CDC), and scientists and researchers. Response options were “A lot,” “Some,” “Not too much,” and “Not at all”.

Emotional support was measured by asking: “Approximately how many people in your social circle could you talk to if you had a problem, felt sad or depressed?” Response options ranged from “none” to “11 or more.” For analysis, responses were dichotomized as having no one versus at least one person available to capture the practical distinction between having any confiding relationship versus having none, consistent with many prior service-use studies [25], [26].

Covariates included race, Hispanic ethnicity, Middle Eastern or North African (MENA) ethnicity, nativity, gender identity, age, education level, household income, and insurance type. Race was reported from a predefined list (White, Black/African American, Asian American, Native American, Pacific Islander, Other). Respondents selecting more than one race or “Other” were categorized as Multiracial/Other. Native American and Pacific Islander respondents were collapsed into a single category for analysis. Participants reported gender identity by selecting male, female, genderqueer, or other. For analysis, responses of genderqueer or other were combined and classified as transgender/nonbinary (TGNB). Respondents were asked to identify whether their insurance type was under Medicare, Medicaid, or neither of these, dichotomized as receiving Medicare/Medicaid or neither.

### 2.3 Statistical analysis

Analyses were restricted to respondents with elevated mental health symptoms (PHQ-9 ≥10 and/or GAD-2 ≥3). Weighted logistic regression models were fit using the *survey* package in R (version 4.3.2) [27], with interlocking post-stratification weights to better approximate the U.S. adult population by sociodemographic characteristics (including age, race and ethnicity, gender, education, and income level) based on the 2019 American Community Survey data. This is a standard approach for analyzing nonprobability internet samples [28].

Primary outcomes were no current treatment, no current antidepressant use, and no current psychotherapy. All models included sociodemographic covariates. Odds ratios, 95 % confidence intervals, and two-sided p-values are reported, with significance defined as p < .05. To assess whether trust in healthcare institutions and social support account for any portion of observed disparities by race/ethnicity, nativity, gender, age group, and insurance type, we compared base models (sociodemographic variables only) with models that additionally adjusted for each trust and social support variable separately.

## 3 Results

The analytic sample included 9733 respondents with elevated depressive and/or anxiety symptoms (PHQ-9 ≥10 and/or GAD-2 ≥3). 5676 (58.3 %) identified as female, 3917 (40.3 %) as male, and 138 (1.4 %) as transgender or gender nonbinary (TGNB). By race and ethnicity, 326 (3.3 %) were Asian American, 1449 (14.9 %) Black, 1320 (13.6 %) Hispanic, 835 (8.6 %) Multiracial/Other, and 6900 (70.9 %) White. Additional sample characteristics are presented in Table 1, including both unweighted and weighted counts. Among respondents with elevated symptoms, a majority - 6589 (66.3 %) - were not receiving antidepressant medication or psychotherapy. Treatment prevalence across sociodemographic subgroups is summarized in Supplemental Table 1.

**Table 1:** Sample characteristics of respondents with elevated depressive and/or anxiety symptoms (PHQ-9 ≥10 and/or GAD-2 ≥3).

**Category**	**Variable**	**N (Weighted)**	**Percent/Mean (SD)**
**Total Elevated Symptoms Sample**	N	9733 (9960)	100.0 (100.0)%
**Race/Ethnicity**	White	6900 (7019)	70.9 (70.5)%
	Black	1449 (1305)	14.9 (13.1)%
	Asian American	326 (498)	3.3 (5)%
	Native American/Pacific Islander	223 (201)	2.3 (2)%
	Multiracial/Other	835 (936)	8.6 (9.4)%
**Ethnicity**	Non-Hispanic	8413 (8251)	86.4 (82.8)%
	Hispanic	1320 (1709)	13.6 (17.2)%
	Non-MENA	9348 (9560)	96 (96)%
	Middle Eastern or North African	385 (400)	4 (4)%
**Nativity**	US-born	9333 (9486)	95.9 (95.2)%
	Foreign-born	400 (474)	4.1 (4.8)%
**Gender**	Female	5676 (5423)	58.3 (54.5)%
	Male	3917 (4372)	40.3 (43.9)%
	TGNB	138 (162)	1.4 (1.6)%
**Age Group**	45–64	2879 (2881)	29.6 (28.9)%
	65 and over	768 (806)	7.9 (8.1)%
	25–44	4730 (4380)	48.6 (44)%
	18–24	1356 (1892)	13.9 (19)%
**Education Level**	College Degree	2710 (1719)	27.8 (17.3)%
	Graduate Degree	860 (936)	8.8 (9.4)%
	Some College	2508 (3214)	25.8 (32.3)%
	High School Graduate	3120 (3490)	32.1 (35)%
	Some High School or Less	535 (601)	5.5 (6)%
**Household Income**	100 K+	1459 (1457)	15 (14.6)%
	75–100 K	971 (1029)	10 (10.3)%
	50–75 K	1734 (1757)	17.8 (17.6)%
	25–50 K	2675 (2741)	27.5 (27.5)%
	Under 25 K	2893 (2975)	29.7 (29.9)%
**Insurance Type**	Private/Other	4013 (4125)	41.2 (41.4)%
	Medicare/Medicaid	5720 (5835)	58.8 (58.6)%
**Trust in Doctors**	A lot	3306 (3509)	34.1 (35.4)%
	Some	4706 (4721)	48.5 (47.6)%
	Not too much	1274 (1294)	13.1 (13)%
	Not at all	414 (399)	4.3 (4)%
**Trust in Pharmaceutical Companies**	A lot	1278 (1377)	13.1 (13.8)%
	Some	3474 (3645)	35.7 (36.6)%
	Not too much	3169 (3167)	32.6 (31.8)%
	Not at all	1798 (1758)	18.5 (17.7)%
**Trust in CDC**	A lot	2263 (2352)	23.3 (23.7)%
	Some	3718 (3844)	38.3 (38.7)%
	Not too much	2111 (2139)	21.7 (21.5)%
	Not at all	1620 (1606)	16.7 (16.2)%
**Trust in Science**	A lot	3244 (3374)	33.4 (34)%
	Some	4497 (4570)	46.3 (46)%
	Not too much	1503 (1535)	15.5 (15.4)%
	Not at all	465 (455)	4.8 (4.6)%
**Emotional Social Support**	Has emotional support (1 + people)	8339 (8523)	86.3 (86.2)%
	No emotional support (0 people)	1324 (1364)	13.7 (13.8)%
**PHQ-9 Score**	Mean (SD)	9618 (9846)	14.3 (5.7) [14.3 (5.6)]
**GAD-2 Score**	Mean (SD)	9703 (9931)	3.8 (1.6) [3.8 (1.7)]

We first fit a base survey-weighted multivariate logistic regression model including all sociodemographic predictors to estimate odds ratios for the likelihood of being untreated (adjusted for all sociodemographic variables). Compared with White respondents, Asian American (OR = 2.04, 95 % CI 1.49–2.78), Black (OR = 1.94, 95 % CI 1.66–2.27), Hispanic (OR = 1.41, 95 % CI 1.19–1.67), and Multiracial/Other (OR = 1.28, 95 % CI 1.05–1.57) respondents all had significantly higher odds of reporting no current treatment. Foreign-born respondents were also more likely to be untreated (OR = 1.43, 95 % CI 1.08–1.9). Patterns also differed by gender, with men more likely to be untreated than women (OR = 1.7, 95 % CI 1.53–1.89), while TGNB respondents had the lowest odds of treatment absence (OR = 0.38, 95 % CI 0.25–0.57). Age differences were also apparent, with young adults aged 18–24 (OR = 1.44, 95 % CI 1.21–1.7) and 25–44 (OR = 1.38, 95 % CI 1.23–1.54) more likely to lack treatment compared to adults 45–64 as well as older adults aged 65 + (OR = 1.66, 95 % CI 1.36–2.03). Finally, respondents with Medicare or Medicaid coverage had lower odds of being untreated than those not on public insurance (OR = 0.64, 95 % CI 0.57–0.71). Baseline associations from the model including only sociodemographic characteristics are shown in Supplemental Figure 1.

**Fig. 1:**
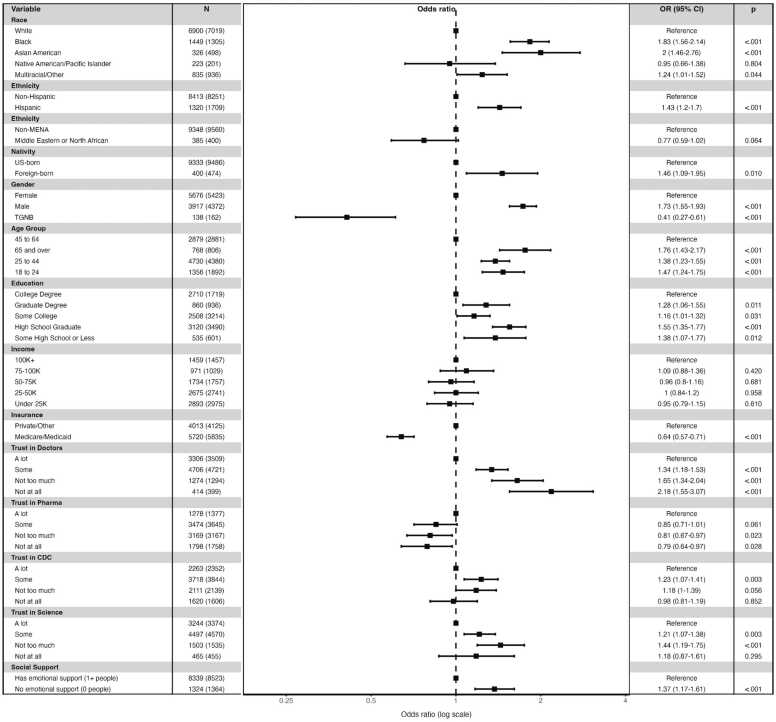
Adjusted odds ratios of being untreated among adults with elevated symptoms, by trust in healthcare institutions, emotional social support, and race/ethnicity, gender, nativity, income, and insurance status.

We then expanded the multivariate model to include trust in healthcare institutions and availability of emotional support (Fig. 1). Lower trust in doctors and hospitals was associated with higher odds of being untreated. Compared with respondents expressing “a lot” of trust, those reporting “some” trust (OR = 1.34, 95 % CI 1.18–1.53), “not too much” trust (OR = 1.65, 95 % CI 1.34–2.04), or “not at all” trust (OR = 2.18, 95 % CI 1.55–3.07) had significantly higher odds of being untreated. Trust in science showed a similar pattern, with those expressing only “some” trust (OR = 1.21, 95 % CI 1.07–1.38) and “not too much” trust (OR = 1.44, 95 % CI 1.19–1.75) having higher odds of being untreated. Notably, unlike other trust indicators, lower trust in pharmaceutical companies was associated with lower odds of lacking any treatment. That is, individuals indicating greater trust in pharmaceutical companies were *more* likely to be lacking mental health treatment. Compared with respondents reporting ‘a lot’ of trust, those with ‘not too much’ (OR = 0.81, 95 % CI 0.67–0.97) or ‘not at all’ (OR = 0.79, 95 % CI 0.64–0.97) trust had significantly lower odds of being untreated. Emotional support was also a strong predictor, as respondents with no one available to talk to when feeling sad or depressed had higher odds of being untreated (OR = 1.37, 95 % CI 1.17–1.61) compared to those with at least one source of support.

Despite these independent associations, inclusion of trust and support measures in regression models produced very minimal changes in the sociodemographic main effects on lack of treatment (Supplemental Table 2), demonstrating that trust factors explained very little of the treatment disparities by race/ethnicity, nativity, gender, age, and insurance status.

**Fig. 2:**
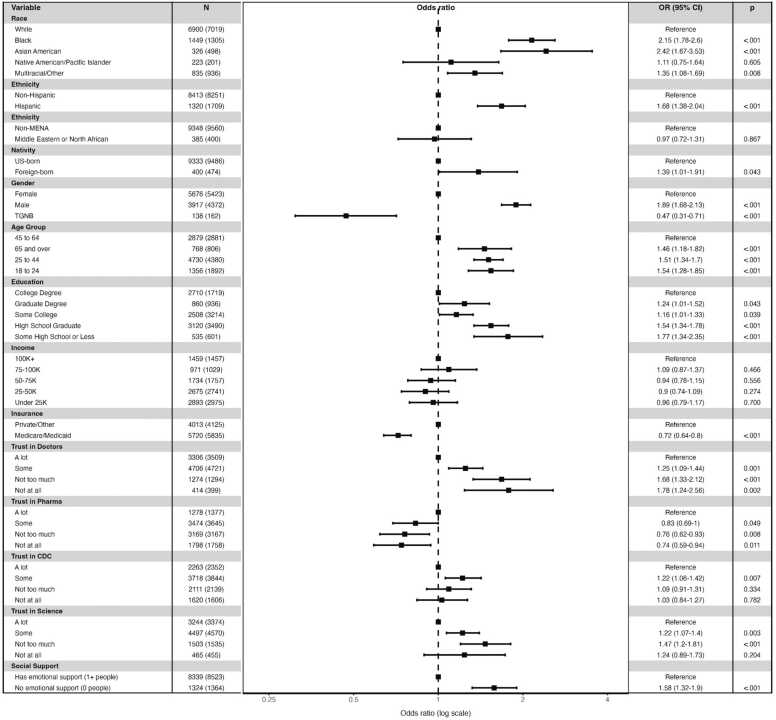
Adjusted odds ratios of not being on an antidepressant among adults with elevated symptoms, by trust in healthcare institutions, emotional social support, and race/ethnicity, gender, nativity, income, and insurance status.

**Fig. 3:**
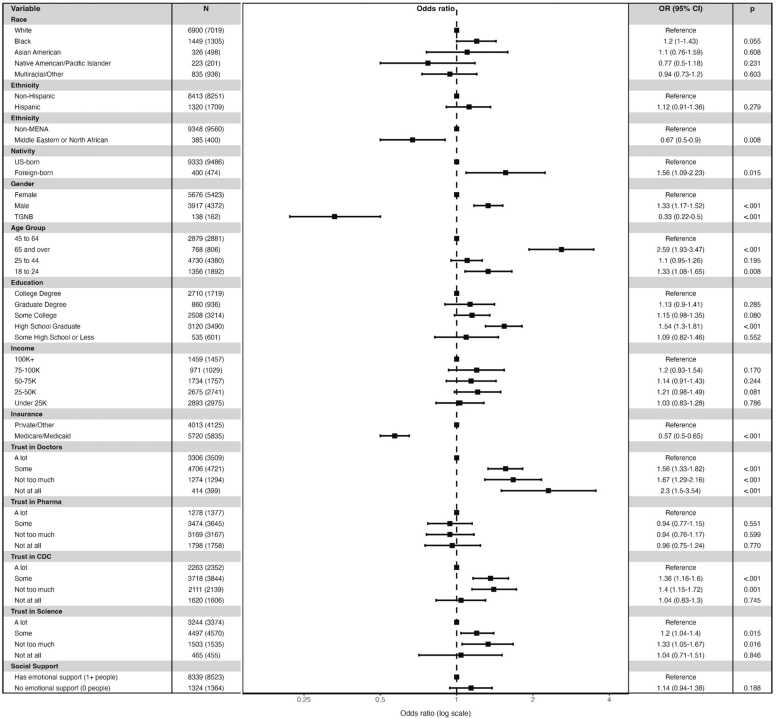
Adjusted odds ratios of not receiving psychotherapy among adults with elevated symptoms, by trust in healthcare institutions, emotional social support, and race/ethnicity, gender, nativity, income, and insurance status.

In secondary analyses, we examined predictors of not taking antidepressants (Fig. 2) and not receiving psychotherapy (Fig. 3) as distinct outcomes among respondents with depressive or anxiety symptoms. Of 9733 respondents, 2546 (26.2 %) reported current antidepressant use and 1770 (18.2 %) reported current psychotherapy (Supplemental Table 2). Black (OR = 2.15, 95 % CI 1.78–2.6), Asian (OR = 2.42, 95 % CI 1.67–3.53), Hispanic (OR = 1.68, 95 % CI 1.38–2.04), and Multiracial/Other adults (OR = 1.35, 95 % CI 1.08–1.69) were significantly more likely than White respondents to not be on an antidepressant. Conversely, racial groups did not differ significantly in psychotherapy use. Foreign-born adults showed elevated odds of not receiving psychotherapy (OR = 1.56, 95 % CI 1.09–2.23) or antidepressants (OR = 1.39, 95 % CI 1.01–1.91). Younger adults (18–44) had greater likelihood of antidepressant non-use (18–24: OR = 1.54, 95 % CI 1.28–1.85; 25–44: OR = 1.51, 95 % CI 1.34–1.7), and 18–24-year-olds had greater likelihood of lack of psychotherapy (OR = 1.33, 95 % CI 1.08–1.65). Adults ≥ 65 were significantly more likely to not be receiving therapy (OR = 2.59, 95 % CI 1.93–3.47) or antidepressants (OR = 1.46, 95 % CI 1.18–1.82).

We next examined main effects, and moderating effects, of trust. Low trust in doctors/hospitals was associated with psychotherapy non-use (e.g., “not at all” trust: OR = 2.3, 95 % CI 1.5–3.54) and antidepressant non-use (e.g., “not at all” trust: OR = 1.78, 95 % CI 1.24–2.56). Low trust in pharmaceutical companies was conversely associated with lower odds of antidepressant non-use (e.g., “not at all”: OR = 0.74, 95 % CI 0.59–0.94) and was not associated with psychotherapy use. Low trust in the CDC predicted psychotherapy non-use for those with “not too much” (OR = 1.4, 95 % CI 1.15–1.72) and “some” trust (OR- 1.36, 95 % CI 1.16–1.6). Low trust in science was associated with non-use of both treatment types (e.g., “not too much”: antidepressants OR = 1.47, 95 % CI 1.2–1.81; psychotherapy OR = 1.33, 95 % CI 1.05–1.67). Similar to models for lack of overall treatment, inclusion of each trust and social support variable produced minimal change in the sociodemographic associations for both antidepressant (Supplemental Table 3) and psychotherapy (Supplemental Table 4) non-use.

## 4 Discussion

In this national sample of adults reporting moderate-to-severe depressive and/or anxiety symptoms, nearly two thirds were not receiving any current mental health treatment, and treatment gaps between socioeconomic groups were pronounced. Black, Hispanic, Asian, Multiracial, and foreign-born adults were significantly more likely than White and U.S.-born adults to be untreated. Men, younger adults, and those without public insurance also had higher odds of going without treatment.

Prior national surveys have described mental health treatment disparities in the general population [29], [30], but most did not restrict analyses to adults experiencing elevated symptom burden, leaving unclear whether lower treatment rates in some sociodemographic groups reflect fewer individuals experiencing reported mental health burden or lower treatment use among those who do. A few earlier studies using pre–Covid-19 data documented racial and ethnic differences in treatment among adults with psychological distress [31], [32], [33], [34]; however, little work has examined these patterns recently, despite significant increases in both depressive/anxious symptom prevalence and overall mental health service use since the Covid-19 pandemic, with notable changes among racial and ethnic minority groups [35], [36]. Our findings extend prior work by demonstrating that pronounced disparities in mental health treatment utilization persist post-pandemic even when analyses are restricted to those who are acutely symptomatic.

Trust in health care institutions and the availability of emotional support each independently predicted treatment use. Adults reporting less trust in physicians and hospitals or less trust in science were more likely to be untreated, and having no one to turn to for emotional support was also associated with lack of mental health care. These patterns are consistent with work showing that institutional mistrust can discourage engagement with health services [12] and that lack of strong social networks can discourage care-seeking [17], [37]. Conversely, our finding that trust in pharmaceutical companies was inversely related to lack of treatment, particularly for antidepressant use, is notable. Although we could not identify prior work directly examining this facet of healthcare system trust and treatment uptake, we hypothesize that our results may suggest that at least a subset of those currently symptomatic treated with medications may have more negative attitudes toward the pharmaceutical industry due to greater exposure to prescription processes and medication management. Having no one to confide in about emotional troubles also predicted lack of mental health care, suggesting that social networks play a role in facilitating mental health care initiation by encouraging formal help-seeking [16], [17].

In secondary analyses focused on each treatment type separately, several sociodemographic predictors of antidepressant non-use emerged, including higher odds among Black, Asian, Hispanic, and Multiracial/Other adults, younger adults, and those ≥ 65. Psychotherapy non-use was most strongly associated with foreign-born status and older age, with no significant racial or ethnic differences. Public insurance coverage was linked to greater likelihood of receiving both treatments. Low trust in doctors and hospitals was associated with non-use of both antidepressants and psychotherapy, whereas low trust in pharmaceutical companies related only to antidepressant non-use, and low trust in the CDC predicted psychotherapy non-use. Although we did not formally compare the two outcomes, the absence of significant racial and ethnic differences in psychotherapy use may reflect previously reported preferences among racial and ethnic minority adults for therapy/counseling when seeking mental health care [38], [39]. Future work should directly examine sociodemographic differences in single-modality treatment choices.

Notably, adjustment for trust and social support produced minimal changes in the association between sociodemographic characteristics and treatment absence. Several explanations are plausible. First, structural barriers such as cost, insurance design, provider shortages, biases in prescribing [40], discriminatory treatment [41], as well as attitudinal barriers including stigma [42], lower perceived need for services [43], [44], or limited access to mental health literacy [45], [46], may be more important drivers of sociodemographic disparities than the healthcare trust and social support factors we measured. Additionally, our brief single-item trust and support measures may capture general sentiment but not the culturally grounded, context-specific mistrust that shapes minority communities’ interactions with care.

### 4.1 Limitations

This study has multiple limitations. First, the cross-sectional design precludes causal inference, and we cannot determine whether trust or social support precede treatment decisions. The web-based, nonprobability sampling strategy we employ may generalize less well than probability samples, although weighting helps approximate the U.S. adult population and multiple prior studies applying this methodology demonstrate convergent validity either with administrative data, or probability sampling [20], [28]. Furthermore, all measures, including mental health symptoms, treatment use, trust, and support, were self-reported, and we applied common screening thresholds rather than clinical diagnoses. While our psychotherapy measure captured formal talk therapy, it did not capture potential engagement in group-based, pastoral, marital, substance-use, or other supportive counseling services. We were also unable to assess receipt of other evidence-based interventions (e.g., neuromodulation or light therapy). Furthermore, because symptom thresholds do not equate to treatment indication, lack of treatment in this sample does not necessarily imply inappropriate care. Finally, while we examined whether trust and support accounted for sociodemographic disparities in treatment use, these analyses do not constitute a formal mediation analysis or rule out the influence of other barriers.

### 4.2 Conclusion

Among U.S. adults with moderate-to-severe depressive or anxiety symptoms, racial and ethnic minority groups, men, and those born outside the United States were less likely to receive any mental health treatment than their respective comparison groups. Trust in health care and social support were associated with likelihood of treatment but explained little of the persistent sociodemographic differences. Future work should examine alternative potential mediators, including structural barriers, stigma, or different forms of healthcare mistrust to clarify how they shape help-seeking over time and interact with disparities.

## Declaration of Competing Interest

The authors declare the following financial interests/personal relationships which may be considered as potential competing interests: Roy Perlis reports financial support was provided by National Institute of Mental Health. David Lazer reports financial support was provided by National Institute of Mental Health. Katherine Ognyanova reports financial support was provided by National Science Foundation. David Lazer reports financial support was provided by National Science Foundation. James N. Druckman reports financial support was provided by National Science Foundation. Matthew A. Baum reports financial support was provided by National Science Foundation. David Lazer reports financial support was provided by John S and James L Knight Foundation. David Lazer reports financial support was provided by Peter G Peterson Foundation. Roy Perlis reports a relationship with Genomind Inc that includes: consulting or advisory. Roy Perlis reports a relationship with Circular Genomics that includes: consulting or advisory and equity or stocks. Roy Perlis reports a relationship with Alkermes Inc that includes: consulting or advisory. Corresponding author, Dr. Roy Perlis, is the Editor-in-Chief of JAMA+ AI and a paid Associate Editor for JAMA Network. If there are other authors, they declare that they have no known competing financial interests or personal relationships that could have appeared to influence the work reported in this paper.

## Footnotes

Appendix A Supplementary data associated with this article can be found in the online version at doi:10.1016/j.xjmad.2026.100166.
