# Supplementary material for: Sociodemographic disparities, healthcare system trust, and social support in mental health treatment among U.S. adults with depressive or anxiety symptoms
Source: J Mood Anxiety Disord. 2026 Jan 13;13:100166. doi: 10.1016/j.xjmad.2026.100166 (PMC12854058; doi:10.1016/j.xjmad.2026.100166)
Supplement: Supplementary file 1 — Supplementary material [file mmc1.docx]

Supplementary Materials

[Supplemental Figure 1: Adjusted odds ratios of being untreated among adults with elevated symptoms, by sociodemographic factors only 2](#_Toc211099887)

[Supplemental Table 1: Prevalence of current antidepressant use, psychotherapy use, and any treatment among adults with elevated symptoms, stratified by sociodemographic subgroup 3](#_Toc211099889)

[Supplementary Table 2: Association between sociodemographic characteristics and being untreated, before and after adjustment for trust and emotional support 5](#_Toc211099890)

[Supplementary Table 3: Association between sociodemographic characteristics and not being on antidepressants, before and after adjustment for trust and emotional support 9](#_Toc211099891)

[Supplementary Table 4: Association between sociodemographic characteristics and not being in therapy, before and after adjustment for trust and emotional support 13](#_Toc211099892)

# **Supplemental Figure 1:** Adjusted odds ratios of being untreated among adults with elevated symptoms, by sociodemographic factors only

#
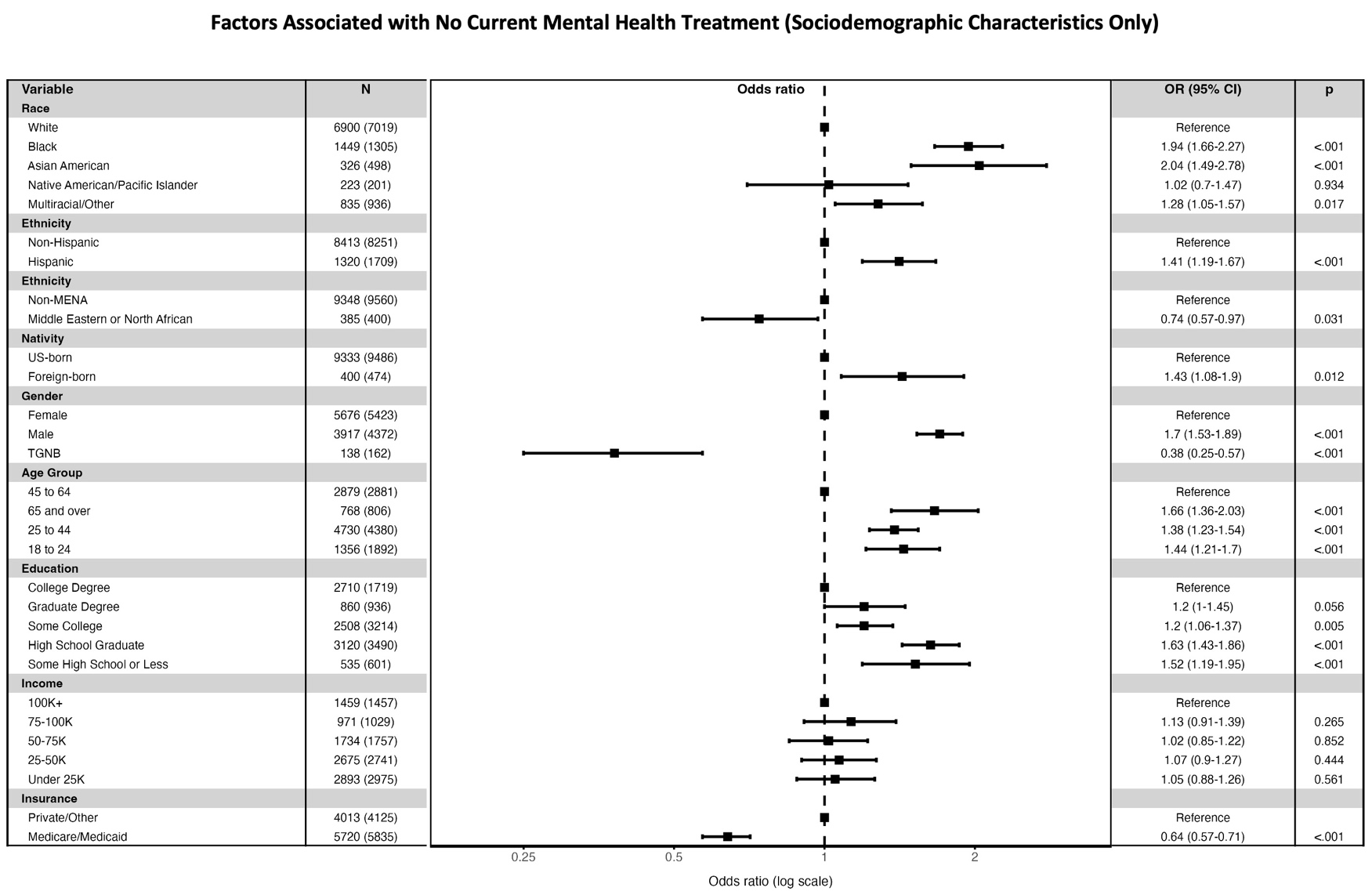


# **Supplemental Table 1**: Prevalence of current antidepressant use, psychotherapy use, and any treatment among adults with elevated symptoms, stratified by sociodemographic subgroup

| **Category** | **Subgroup** | **N (weighted)** | **Antidepressant Use** | **Psychotherapy Use** | **Any Treatment** |
| --- | --- | --- | --- | --- | --- |
| **Overall** | All participants | 9733 (9960) | 26.2% (24.6%) [2546 (2453)] | 18.2% (17.6%) [1770 (1753)] | 33.7% (32.2%) [3284 (3212)] |
| **Race** | White | 6900 (7019) | 29.9% (28.4%) [2063 (1995)] | 18.7% (18%) [1288 (1266)] | 37.1% (35.7%) [2561 (2507)] |
|  | Black | 1449 (1305) | 14.1% (14.3%) [205 (186)] | 15.6% (15.4%) [226 (201)] | 22.5% (22.2%) [326 (290)] |
|  | Asian American | 326 (498) | 13.2% (12.9%) [43 (64)] | 15.6% (15.9%) [51 (79)] | 21.5% (20.7%) [70 (103)] |
|  | Native American/Pacific Islander | 223 (201) | 22.9% (20.9%) [51 (42)] | 19.3% (20.9%) [43 (42)] | 31.8% (31.8%) [71 (64)] |
|  | Multiracial/Other | 835 (936) | 22% (17.6%) [184 (165)] | 19.4% (17.7%) [162 (166)] | 30.7% (26.5%) [256 (248)] |
| **Ethnicity** | Non-Hispanic | 8413 (8251) | 27.3% (26.4%) [2298 (2181)] | 18.2% (17.8%) [1527 (1470)] | 34.7% (33.8%) [2918 (2785)] |
|  | Hispanic | 1320 (1709) | 18.8% (15.9%) [248 (272)] | 18.4% (16.6%) [243 (283)] | 27.7% (25%) [366 (427)] |
|  | Non-MENA | 9348 (9560) | 26.4% (24.8%) [2467 (2373)] | 18% (17.3%) [1681 (1655)] | 33.8% (32.2%) [3157 (3078)] |
|  | Middle Eastern or North African | 385 (400) | 20.5% (20%) [79 (80)] | 23.1% (24.8%) [89 (99)] | 33% (33.8%) [127 (135)] |
| **Nativity** | US-born | 9333 (9486) | 26.5% (25.1%) [2476 (2381)] | 18.4% (17.9%) [1713 (1695)] | 34.2% (32.8%) [3188 (3110)] |
|  | Foreign-born | 400 (474) | 17.5% (15.2%) [70 (72)] | 14.2% (12.4%) [57 (59)] | 24% (21.5%) [96 (102)] |
| **Gender** | Female | 5676 (5423) | 30.4% (29.4%) [1728 (1592)] | 19% (18.6%) [1079 (1007)] | 38% (36.9%) [2157 (2001)] |
|  | Male | 3917 (4372) | 19.4% (18.1%) [761 (791)] | 16.3% (15.6%) [640 (680)] | 26.9% (25.6%) [1054 (1118)] |
|  | TGNB | 138 (162) | 41.3% (42.6%) [57 (69)] | 36.2% (39.5%) [50 (64)] | 52.2% (55.6%) [72 (90)] |
| **Age Group** | 45 to 64 | 2879 (2881) | 32.5% (30.4%) [935 (876)] | 19.8% (19.4%) [569 (559)] | 40.1% (38%) [1154 (1094)] |
|  | 65 and over | 768 (806) | 30.5% (29.4%) [234 (237)] | 12.5% (11.3%) [96 (91)] | 34.2% (33%) [263 (266)] |
|  | 25 to 44 | 4730 (4380) | 23.1% (21.8%) [1092 (955)] | 18.7% (18.4%) [885 (804)] | 31.2% (30.1%) [1477 (1320)] |
|  | 18 to 24 | 1356 (1892) | 21% (20.3%) [285 (385)] | 16.2% (15.8%) [220 (299)] | 28.8% (28.1%) [390 (532)] |
| **Education Level** | College Degree | 2710 (1719) | 29.4% (29.5%) [798 (507)] | 20.3% (20.2%) [549 (348)] | 37.6% (37.4%) [1018 (643)] |
|  | Graduate Degree | 860 (936) | 26.2% (25.9%) [225 (242)] | 22.1% (21.8%) [190 (204)] | 34.7% (33.8%) [298 (316)] |
|  | Some College | 2508 (3214) | 28.4% (27%) [712 (867)] | 18.2% (18%) [457 (580)] | 35.6% (34.4%) [893 (1107)] |
|  | High School Graduate | 3120 (3490) | 22.5% (20.7%) [701 (724)] | 15.1% (14.3%) [471 (500)] | 29.2% (27.5%) [910 (961)] |
|  | Some High School or Less | 535 (601) | 20.6% (19%) [110 (114)] | 19.3% (20.1%) [103 (121)] | 30.8% (30.9%) [165 (186)] |
| **Household Income** | 100K+ | 1459 (1457) | 26.4% (24.6%) [385 (358)] | 21.5% (20.9%) [313 (304)] | 34.9% (32.9%) [509 (480)] |
|  | 75-100K | 971 (1029) | 24.1% (21.5%) [234 (221)] | 17.4% (16.2%) [169 (167)] | 31.5% (29%) [306 (298)] |
|  | 50-75K | 1734 (1757) | 26.2% (24.6%) [455 (432)] | 16.4% (16.1%) [285 (283)] | 33.3% (31.9%) [578 (561)] |
|  | 25-50K | 2675 (2741) | 27% (26.1%) [723 (715)] | 16.6% (15.9%) [443 (437)] | 33.4% (32%) [893 (878)] |
|  | Under 25K | 2893 (2975) | 25.9% (24.4%) [749 (727)] | 19.4% (18.9%) [560 (561)] | 34.5% (33.4%) [998 (994)] |
| **Insurance Type** | Private/Other | 4013 (4125) | 23.3% (21.6%) [934 (891)] | 14.3% (13.7%) [572 (564)] | 29.3% (27.5%) [1174 (1136)] |
|  | Medicare/Medicaid | 5720 (5835) | 28.2% (26.8%) [1612 (1561)] | 20.9% (20.4%) [1198 (1189)] | 36.9% (35.6%) [2110 (2076)] |
| **Trust in Doctors** | A lot | 3306 (3509) | 30.2% (28.5%) [999 (999)] | 23.4% (23.2%) [775 (814)] | 39.2% (37.6%) [1296 (1321)] |
|  | Some | 4706 (4721) | 26.1% (24.3%) [1226 (1149)] | 16.4% (15.1%) [770 (712)] | 33.1% (31.1%) [1558 (1469)] |
|  | Not too much | 1274 (1294) | 18.4% (17.5%) [235 (226)] | 13.4% (13.4%) [171 (173)] | 25.3% (24.8%) [322 (321)] |
|  | Not at all | 414 (399) | 17.4% (17.3%) [72 (69)] | 12.1% (12.5%) [50 (50)] | 22.5% (22.6%) [93 (90)] |
| **Trust in Pharmaceutical Companies** | A lot | 1278 (1377) | 25.4% (23.4%) [325 (322)] | 22.5% (22.1%) [287 (305)] | 34.4% (32.9%) [440 (453)] |
|  | Some | 3474 (3645) | 27% (25.2%) [939 (918)] | 19.2% (18.3%) [668 (666)] | 35% (33.2%) [1216 (1209)] |
|  | Not too much | 3169 (3167) | 25.9% (25%) [820 (793)] | 16.4% (16.2%) [519 (512)] | 32.7% (31.8%) [1036 (1008)] |
|  | Not at all | 1798 (1758) | 25.5% (23.7%) [458 (416)] | 16.3% (15.2%) [293 (268)] | 32.6% (30.5%) [587 (537)] |
| **Trust in CDC** | A lot | 2263 (2352) | 30.8% (29.4%) [697 (691)] | 24.5% (24%) [554 (565)] | 39.9% (38.4%) [902 (902)] |
|  | Some | 3718 (3844) | 26% (23.8%) [966 (914)] | 17.3% (16.3%) [642 (627)] | 33.5% (31.2%) [1245 (1201)] |
|  | Not too much | 2111 (2139) | 23.8% (22.9%) [503 (489)] | 14.1% (13.7%) [298 (293)] | 29.7% (28.8%) [628 (616)] |
|  | Not at all | 1620 (1606) | 23.3% (22.3%) [377 (358)] | 16.9% (16.6%) [273 (266)] | 31.1% (30.5%) [504 (490)] |
| **Trust in Science** | A lot | 3244 (3374) | 30.8% (29.5%) [1000 (995)] | 22.7% (22.5%) [737 (758)] | 39.2% (38.1%) [1273 (1285)] |
|  | Some | 4497 (4570) | 25.4% (23.7%) [1142 (1083)] | 16.5% (15.7%) [744 (716)] | 32.7% (30.9%) [1469 (1413)] |
|  | Not too much | 1503 (1535) | 19.9% (18.3%) [299 (281)] | 14% (13.1%) [211 (201)] | 26.6% (24.9%) [400 (382)] |
|  | Not at all | 465 (455) | 20.4% (19.1%) [95 (87)] | 15.3% (15.4%) [71 (70)] | 28% (26.8%) [130 (122)] |
| **Social Support** | Has emotional support (1+ people) | 8339 (8523) | 27.4% (25.9%) [2284 (2206)] | 18.7% (18.1%) [1559 (1542)] | 35% (33.5%) [2919 (2854)] |
|  | No emotional support (0 people) | 1324 (1364) | 17.9% (16.4%) [237 (224)] | 14.5% (14.1%) [192 (193)] | 25.4% (24.3%) [336 (331)] |

# **Supplementary Table 2:** Association between sociodemographic characteristics and being untreated, before and after adjustment for trust and emotional support

*†Model 1: Adjusted for all sociodemographic characteristics (race, ethnicity, nativity, gender, age, education, income, insurance)*

*‡Model 2: Model 1 + specified trust/social support variable*

| **Category** | **Subgroup** | **N (Weighted)** | **Added Variable** | **Unadjusted** | **†Model 1** | **‡Model 2** |
| --- | --- | --- | --- | --- | --- | --- |
| **Race** | White | 6877 (6994) | Trust in Doctors | 1.00 (Reference) | 1.00 (Reference) | 1.00 (Reference) |
|  | Black | 1444 (1299) | Trust in Doctors | 1.94 (1.66-2.25) | 1.93 (1.65-2.26) | 1.88 (1.61-2.2) |
|  | Asian American | 326 (498) | Trust in Doctors | 2.14 (1.58-2.88) | 2.04 (1.49-2.78) | 2.01 (1.48-2.74) |
|  | Native American/Pacific Islander | 222 (200) | Trust in Doctors | 1.16 (0.82-1.64) | 1.01 (0.7-1.46) | 0.98 (0.68-1.41) |
|  | Multiracial/Other | 831 (932) | Trust in Doctors | 1.53 (1.27-1.84) | 1.27 (1.04-1.56) | 1.25 (1.02-1.54) |
| **Ethnicity** | Non-Hispanic | 8383 (8219) | Trust in Doctors | 1.00 (Reference) | 1.00 (Reference) | 1.00 (Reference) |
|  | Hispanic | 1317 (1705) | Trust in Doctors | 1.52 (1.31-1.78) | 1.41 (1.19-1.67) | 1.43 (1.21-1.7) |
|  | Non-MENA | 9315 (9523) | Trust in Doctors | 1.00 (Reference) | 1.00 (Reference) | 1.00 (Reference) |
|  | Middle Eastern or North African | 385 (400) | Trust in Doctors | 0.94 (0.73-1.21) | 0.74 (0.57-0.98) | 0.77 (0.59-1) |
| **Nativity** | US-born | 9301 (9450) | Trust in Doctors | 1.00 (Reference) | 1.00 (Reference) | 1.00 (Reference) |
|  | Foreign-born | 399 (473) | Trust in Doctors | 1.78 (1.36-2.33) | 1.43 (1.08-1.89) | 1.43 (1.08-1.9) |
| **Gender** | Female | 5654 (5397) | Trust in Doctors | 1.00 (Reference) | 1.00 (Reference) | 1.00 (Reference) |
|  | Male | 3906 (4361) | Trust in Doctors | 1.7 (1.54-1.88) | 1.7 (1.53-1.89) | 1.76 (1.58-1.96) |
|  | TGNB | 138 (162) | Trust in Doctors | 0.46 (0.31-0.69) | 0.38 (0.25-0.57) | 0.38 (0.26-0.57) |
| **Age Group** | 45 to 64 | 2866 (2869) | Trust in Doctors | 1.00 (Reference) | 1.00 (Reference) | 1.00 (Reference) |
|  | 65 and over | 761 (795) | Trust in Doctors | 1.24 (1.02-1.51) | 1.65 (1.35-2.03) | 1.74 (1.42-2.14) |
|  | 25 to 44 | 4719 (4369) | Trust in Doctors | 1.42 (1.27-1.58) | 1.37 (1.23-1.54) | 1.38 (1.23-1.54) |
|  | 18 to 24 | 1354 (1890) | Trust in Doctors | 1.56 (1.33-1.84) | 1.43 (1.21-1.7) | 1.47 (1.24-1.74) |
| **Education** | College Degree | 2706 (1717) | Trust in Doctors | 1.00 (Reference) | 1.00 (Reference) | 1.00 (Reference) |
|  | Graduate Degree | 857 (933) | Trust in Doctors | 1.17 (0.98-1.39) | 1.2 (0.99-1.44) | 1.29 (1.07-1.55) |
|  | Some College | 2498 (3204) | Trust in Doctors | 1.14 (1-1.29) | 1.2 (1.05-1.37) | 1.18 (1.03-1.34) |
|  | High School Graduate | 3106 (3471) | Trust in Doctors | 1.56 (1.38-1.76) | 1.62 (1.42-1.85) | 1.6 (1.4-1.83) |
|  | Some High School or Less | 533 (599) | Trust in Doctors | 1.32 (1.05-1.67) | 1.51 (1.18-1.93) | 1.44 (1.12-1.85) |
| **Income** | 100K+ | 1452 (1448) | Trust in Doctors | 1.00 (Reference) | 1.00 (Reference) | 1.00 (Reference) |
|  | 75-100K | 970 (1028) | Trust in Doctors | 1.21 (0.99-1.48) | 1.13 (0.92-1.4) | 1.11 (0.89-1.37) |
|  | 50-75K | 1726 (1749) | Trust in Doctors | 1.06 (0.9-1.25) | 1.03 (0.86-1.24) | 0.98 (0.82-1.18) |
|  | 25-50K | 2666 (2732) | Trust in Doctors | 1.05 (0.9-1.22) | 1.08 (0.91-1.28) | 1.04 (0.87-1.23) |
|  | Under 25K | 2885 (2965) | Trust in Doctors | 0.99 (0.85-1.14) | 1.06 (0.89-1.27) | 1 (0.83-1.2) |
| **Insurance** | Private/Other | 4000 (4113) | Trust in Doctors | 1.00 (Reference) | 1.00 (Reference) | 1.00 (Reference) |
|  | Medicare/Medicaid | 5700 (5810) | Trust in Doctors | 0.69 (0.62-0.76) | 0.64 (0.57-0.71) | 0.65 (0.58-0.72) |
| **Race** | White | 6890 (7009) | Trust in Pharmaceutical Companies | 1.00 (Reference) | 1.00 (Reference) | 1.00 (Reference) |
|  | Black | 1446 (1303) | Trust in Pharmaceutical Companies | 1.96 (1.68-2.28) | 1.95 (1.67-2.28) | 1.97 (1.69-2.31) |
|  | Asian American | 326 (498) | Trust in Pharmaceutical Companies | 2.14 (1.58-2.89) | 2.04 (1.5-2.78) | 2.08 (1.52-2.84) |
|  | Native American/Pacific Islander | 223 (201) | Trust in Pharmaceutical Companies | 1.17 (0.83-1.66) | 1.02 (0.7-1.47) | 1.02 (0.7-1.47) |
|  | Multiracial/Other | 834 (935) | Trust in Pharmaceutical Companies | 1.54 (1.28-1.85) | 1.28 (1.04-1.56) | 1.27 (1.03-1.55) |
| **Ethnicity** | Non-Hispanic | 8400 (8238) | Trust in Pharmaceutical Companies | 1.00 (Reference) | 1.00 (Reference) | 1.00 (Reference) |
|  | Hispanic | 1319 (1708) | Trust in Pharmaceutical Companies | 1.53 (1.31-1.78) | 1.41 (1.19-1.67) | 1.43 (1.21-1.69) |
|  | Non-MENA | 9334 (9546) | Trust in Pharmaceutical Companies | 1.00 (Reference) | 1.00 (Reference) | 1.00 (Reference) |
|  | Middle Eastern or North African | 385 (400) | Trust in Pharmaceutical Companies | 0.94 (0.73-1.21) | 0.74 (0.57-0.97) | 0.75 (0.57-0.99) |
| **Nativity** | US-born | 9320 (9473) | Trust in Pharmaceutical Companies | 1.00 (Reference) | 1.00 (Reference) | 1.00 (Reference) |
|  | Foreign-born | 399 (473) | Trust in Pharmaceutical Companies | 1.78 (1.36-2.33) | 1.43 (1.08-1.89) | 1.43 (1.08-1.89) |
| **Gender** | Female | 5668 (5414) | Trust in Pharmaceutical Companies | 1.00 (Reference) | 1.00 (Reference) | 1.00 (Reference) |
|  | Male | 3911 (4367) | Trust in Pharmaceutical Companies | 1.7 (1.54-1.88) | 1.7 (1.53-1.89) | 1.7 (1.53-1.9) |
|  | TGNB | 138 (162) | Trust in Pharmaceutical Companies | 0.46 (0.31-0.69) | 0.38 (0.25-0.57) | 0.38 (0.25-0.56) |
| **Age Group** | 45 to 64 | 2873 (2876) | Trust in Pharmaceutical Companies | 1.00 (Reference) | 1.00 (Reference) | 1.00 (Reference) |
|  | 65 and over | 766 (803) | Trust in Pharmaceutical Companies | 1.23 (1.01-1.5) | 1.65 (1.34-2.02) | 1.66 (1.35-2.03) |
|  | 25 to 44 | 4724 (4376) | Trust in Pharmaceutical Companies | 1.41 (1.27-1.58) | 1.37 (1.22-1.53) | 1.37 (1.22-1.53) |
|  | 18 to 24 | 1356 (1892) | Trust in Pharmaceutical Companies | 1.56 (1.33-1.83) | 1.43 (1.21-1.7) | 1.45 (1.22-1.72) |
| **Education** | College Degree | 2705 (1716) | Trust in Pharmaceutical Companies | 1.00 (Reference) | 1.00 (Reference) | 1.00 (Reference) |
|  | Graduate Degree | 858 (934) | Trust in Pharmaceutical Companies | 1.17 (0.98-1.39) | 1.2 (0.99-1.44) | 1.22 (1.01-1.48) |
|  | Some College | 2505 (3211) | Trust in Pharmaceutical Companies | 1.14 (1-1.28) | 1.2 (1.05-1.36) | 1.19 (1.04-1.35) |
|  | High School Graduate | 3116 (3485) | Trust in Pharmaceutical Companies | 1.57 (1.39-1.77) | 1.63 (1.42-1.86) | 1.63 (1.43-1.87) |
|  | Some High School or Less | 535 (601) | Trust in Pharmaceutical Companies | 1.33 (1.06-1.68) | 1.52 (1.19-1.95) | 1.53 (1.19-1.95) |
| **Income** | 100K+ | 1452 (1450) | Trust in Pharmaceutical Companies | 1.00 (Reference) | 1.00 (Reference) | 1.00 (Reference) |
|  | 75-100K | 971 (1029) | Trust in Pharmaceutical Companies | 1.2 (0.99-1.47) | 1.13 (0.91-1.39) | 1.12 (0.9-1.38) |
|  | 50-75K | 1733 (1756) | Trust in Pharmaceutical Companies | 1.05 (0.89-1.24) | 1.02 (0.85-1.22) | 1 (0.84-1.2) |
|  | 25-50K | 2671 (2737) | Trust in Pharmaceutical Companies | 1.04 (0.89-1.21) | 1.07 (0.9-1.27) | 1.05 (0.88-1.25) |
|  | Under 25K | 2891 (2973) | Trust in Pharmaceutical Companies | 0.98 (0.84-1.14) | 1.06 (0.88-1.27) | 1.04 (0.87-1.25) |
| **Insurance** | Private/Other | 4007 (4121) | Trust in Pharmaceutical Companies | 1.00 (Reference) | 1.00 (Reference) | 1.00 (Reference) |
|  | Medicare/Medicaid | 5712 (5825) | Trust in Pharmaceutical Companies | 0.69 (0.62-0.76) | 0.64 (0.57-0.71) | 0.65 (0.58-0.72) |
| **Race** | White | 6858 (6972) | Social Support | 1.00 (Reference) | 1.00 (Reference) | 1.00 (Reference) |
|  | Black | 1436 (1296) | Social Support | 1.95 (1.68-2.27) | 1.95 (1.67-2.28) | 1.91 (1.63-2.24) |
|  | Asian American | 317 (488) | Social Support | 2.16 (1.59-2.93) | 2.05 (1.5-2.81) | 2.05 (1.49-2.81) |
|  | Native American/Pacific Islander | 223 (201) | Social Support | 1.17 (0.83-1.65) | 1.02 (0.7-1.47) | 0.99 (0.68-1.44) |
|  | Multiracial/Other | 829 (931) | Social Support | 1.54 (1.28-1.85) | 1.28 (1.04-1.57) | 1.27 (1.03-1.55) |
| **Ethnicity** | Non-Hispanic | 8353 (8191) | Social Support | 1.00 (Reference) | 1.00 (Reference) | 1.00 (Reference) |
|  | Hispanic | 1310 (1696) | Social Support | 1.53 (1.31-1.79) | 1.41 (1.19-1.68) | 1.42 (1.2-1.69) |
|  | Non-MENA | 9283 (9492) | Social Support | 1.00 (Reference) | 1.00 (Reference) | 1.00 (Reference) |
|  | Middle Eastern or North African | 380 (395) | Social Support | 0.94 (0.73-1.22) | 0.74 (0.57-0.98) | 0.76 (0.57-0.99) |
| **Nativity** | US-born | 9271 (9422) | Social Support | 1.00 (Reference) | 1.00 (Reference) | 1.00 (Reference) |
|  | Foreign-born | 392 (465) | Social Support | 1.81 (1.38-2.37) | 1.46 (1.1-1.94) | 1.45 (1.09-1.93) |
| **Gender** | Female | 5640 (5388) | Social Support | 1.00 (Reference) | 1.00 (Reference) | 1.00 (Reference) |
|  | Male | 3883 (4334) | Social Support | 1.69 (1.53-1.87) | 1.69 (1.52-1.88) | 1.67 (1.5-1.85) |
|  | TGNB | 138 (162) | Social Support | 0.46 (0.31-0.69) | 0.38 (0.25-0.57) | 0.38 (0.25-0.57) |
| **Age Group** | 45 to 64 | 2861 (2862) | Social Support | 1.00 (Reference) | 1.00 (Reference) | 1.00 (Reference) |
|  | 65 and over | 763 (801) | Social Support | 1.25 (1.02-1.52) | 1.67 (1.36-2.04) | 1.66 (1.35-2.04) |
|  | 25 to 44 | 4698 (4351) | Social Support | 1.42 (1.27-1.58) | 1.37 (1.23-1.54) | 1.39 (1.24-1.55) |
|  | 18 to 24 | 1341 (1873) | Social Support | 1.56 (1.33-1.83) | 1.43 (1.21-1.7) | 1.45 (1.23-1.72) |
| **Education** | College Degree | 2688 (1706) | Social Support | 1.00 (Reference) | 1.00 (Reference) | 1.00 (Reference) |
|  | Graduate Degree | 853 (927) | Social Support | 1.19 (1-1.41) | 1.21 (1-1.46) | 1.21 (1.01-1.47) |
|  | Some College | 2500 (3201) | Social Support | 1.13 (1-1.28) | 1.19 (1.05-1.36) | 1.19 (1.04-1.35) |
|  | High School Graduate | 3091 (3456) | Social Support | 1.57 (1.39-1.78) | 1.64 (1.43-1.87) | 1.62 (1.42-1.85) |
|  | Some High School or Less | 531 (596) | Social Support | 1.33 (1.06-1.68) | 1.52 (1.19-1.95) | 1.5 (1.17-1.92) |
| **Income** | 100K+ | 1447 (1446) | Social Support | 1.00 (Reference) | 1.00 (Reference) | 1.00 (Reference) |
|  | 75-100K | 961 (1019) | Social Support | 1.2 (0.98-1.47) | 1.13 (0.91-1.4) | 1.11 (0.9-1.38) |
|  | 50-75K | 1724 (1747) | Social Support | 1.04 (0.88-1.23) | 1.02 (0.85-1.22) | 1 (0.83-1.2) |
|  | 25-50K | 2652 (2718) | Social Support | 1.03 (0.89-1.2) | 1.07 (0.9-1.27) | 1.04 (0.87-1.24) |
|  | Under 25K | 2878 (2958) | Social Support | 0.97 (0.83-1.12) | 1.04 (0.87-1.25) | 1 (0.84-1.2) |
| **Insurance** | Private/Other | 3987 (4101) | Social Support | 1.00 (Reference) | 1.00 (Reference) | 1.00 (Reference) |
|  | Medicare/Medicaid | 5676 (5787) | Social Support | 0.69 (0.63-0.77) | 0.64 (0.58-0.71) | 0.64 (0.58-0.72) |
| **Race** | White | 6885 (7005) | Trust in CDC | 1.00 (Reference) | 1.00 (Reference) | 1.00 (Reference) |
|  | Black | 1446 (1302) | Trust in CDC | 1.95 (1.68-2.27) | 1.94 (1.66-2.27) | 1.93 (1.65-2.26) |
|  | Asian American | 325 (498) | Trust in CDC | 2.15 (1.59-2.91) | 2.05 (1.5-2.8) | 2.07 (1.51-2.82) |
|  | Native American/Pacific Islander | 222 (200) | Trust in CDC | 1.18 (0.83-1.67) | 1.02 (0.71-1.48) | 1.01 (0.7-1.45) |
|  | Multiracial/Other | 834 (936) | Trust in CDC | 1.54 (1.28-1.85) | 1.28 (1.04-1.56) | 1.25 (1.02-1.53) |
| **Ethnicity** | Non-Hispanic | 8393 (8233) | Trust in CDC | 1.00 (Reference) | 1.00 (Reference) | 1.00 (Reference) |
|  | Hispanic | 1319 (1708) | Trust in CDC | 1.53 (1.31-1.79) | 1.41 (1.19-1.67) | 1.44 (1.21-1.7) |
|  | Non-MENA | 9329 (9542) | Trust in CDC | 1.00 (Reference) | 1.00 (Reference) | 1.00 (Reference) |
|  | Middle Eastern or North African | 383 (399) | Trust in CDC | 0.94 (0.73-1.21) | 0.75 (0.57-0.98) | 0.75 (0.57-0.98) |
| **Nativity** | US-born | 9314 (9469) | Trust in CDC | 1.00 (Reference) | 1.00 (Reference) | 1.00 (Reference) |
|  | Foreign-born | 398 (472) | Trust in CDC | 1.79 (1.37-2.34) | 1.43 (1.08-1.9) | 1.43 (1.08-1.9) |
| **Gender** | Female | 5669 (5418) | Trust in CDC | 1.00 (Reference) | 1.00 (Reference) | 1.00 (Reference) |
|  | Male | 3903 (4358) | Trust in CDC | 1.7 (1.54-1.88) | 1.7 (1.53-1.89) | 1.72 (1.54-1.91) |
|  | TGNB | 138 (162) | Trust in CDC | 0.46 (0.31-0.69) | 0.38 (0.25-0.57) | 0.4 (0.26-0.59) |
| **Age Group** | 45 to 64 | 2874 (2876) | Trust in CDC | 1.00 (Reference) | 1.00 (Reference) | 1.00 (Reference) |
|  | 65 and over | 764 (803) | Trust in CDC | 1.23 (1.01-1.5) | 1.65 (1.35-2.02) | 1.66 (1.35-2.04) |
|  | 25 to 44 | 4721 (4373) | Trust in CDC | 1.42 (1.27-1.58) | 1.37 (1.22-1.53) | 1.36 (1.21-1.52) |
|  | 18 to 24 | 1353 (1889) | Trust in CDC | 1.57 (1.33-1.84) | 1.43 (1.21-1.7) | 1.45 (1.22-1.71) |
| **Education** | College Degree | 2704 (1715) | Trust in CDC | 1.00 (Reference) | 1.00 (Reference) | 1.00 (Reference) |
|  | Graduate Degree | 857 (932) | Trust in CDC | 1.17 (0.98-1.39) | 1.19 (0.99-1.44) | 1.23 (1.02-1.49) |
|  | Some College | 2505 (3212) | Trust in CDC | 1.14 (1-1.28) | 1.2 (1.05-1.37) | 1.19 (1.04-1.35) |
|  | High School Graduate | 3115 (3485) | Trust in CDC | 1.57 (1.39-1.78) | 1.63 (1.43-1.86) | 1.61 (1.41-1.84) |
|  | Some High School or Less | 531 (598) | Trust in CDC | 1.33 (1.05-1.67) | 1.51 (1.18-1.93) | 1.5 (1.17-1.92) |
| **Income** | 100K+ | 1455 (1453) | Trust in CDC | 1.00 (Reference) | 1.00 (Reference) | 1.00 (Reference) |
|  | 75-100K | 968 (1026) | Trust in CDC | 1.21 (0.99-1.47) | 1.13 (0.91-1.39) | 1.12 (0.9-1.38) |
|  | 50-75K | 1730 (1754) | Trust in CDC | 1.05 (0.89-1.24) | 1.02 (0.85-1.22) | 1 (0.83-1.2) |
|  | 25-50K | 2671 (2737) | Trust in CDC | 1.05 (0.9-1.22) | 1.07 (0.9-1.27) | 1.05 (0.88-1.25) |
|  | Under 25K | 2887 (2970) | Trust in CDC | 0.98 (0.85-1.14) | 1.05 (0.88-1.26) | 1.03 (0.86-1.23) |
| **Insurance** | Private/Other | 4006 (4118) | Trust in CDC | 1.00 (Reference) | 1.00 (Reference) | 1.00 (Reference) |
|  | Medicare/Medicaid | 5706 (5823) | Trust in CDC | 0.69 (0.62-0.76) | 0.64 (0.57-0.71) | 0.64 (0.58-0.72) |
| **Race** | White | 6882 (6999) | Trust in Science | 1.00 (Reference) | 1.00 (Reference) | 1.00 (Reference) |
|  | Black | 1445 (1302) | Trust in Science | 1.94 (1.67-2.26) | 1.94 (1.66-2.26) | 1.88 (1.61-2.2) |
|  | Asian American | 325 (496) | Trust in Science | 2.12 (1.57-2.86) | 2.02 (1.48-2.75) | 2.04 (1.49-2.78) |
|  | Native American/Pacific Islander | 223 (201) | Trust in Science | 1.17 (0.83-1.65) | 1.01 (0.7-1.47) | 0.97 (0.67-1.41) |
|  | Multiracial/Other | 834 (935) | Trust in Science | 1.53 (1.28-1.85) | 1.27 (1.04-1.56) | 1.26 (1.03-1.54) |
| **Ethnicity** | Non-Hispanic | 8394 (8229) | Trust in Science | 1.00 (Reference) | 1.00 (Reference) | 1.00 (Reference) |
|  | Hispanic | 1315 (1704) | Trust in Science | 1.53 (1.31-1.78) | 1.41 (1.19-1.68) | 1.4 (1.18-1.66) |
|  | Non-MENA | 9325 (9534) | Trust in Science | 1.00 (Reference) | 1.00 (Reference) | 1.00 (Reference) |
|  | Middle Eastern or North African | 384 (399) | Trust in Science | 0.94 (0.73-1.22) | 0.75 (0.57-0.98) | 0.76 (0.58-1) |
| **Nativity** | US-born | 9310 (9460) | Trust in Science | 1.00 (Reference) | 1.00 (Reference) | 1.00 (Reference) |
|  | Foreign-born | 399 (473) | Trust in Science | 1.78 (1.36-2.33) | 1.43 (1.08-1.89) | 1.45 (1.09-1.92) |
| **Gender** | Female | 5662 (5409) | Trust in Science | 1.00 (Reference) | 1.00 (Reference) | 1.00 (Reference) |
|  | Male | 3908 (4359) | Trust in Science | 1.7 (1.54-1.89) | 1.71 (1.53-1.9) | 1.72 (1.55-1.92) |
|  | TGNB | 137 (161) | Trust in Science | 0.47 (0.31-0.7) | 0.38 (0.26-0.57) | 0.41 (0.28-0.62) |
| **Age Group** | 45 to 64 | 2874 (2877) | Trust in Science | 1.00 (Reference) | 1.00 (Reference) | 1.00 (Reference) |
|  | 65 and over | 764 (800) | Trust in Science | 1.25 (1.02-1.52) | 1.67 (1.36-2.04) | 1.71 (1.4-2.11) |
|  | 25 to 44 | 4719 (4369) | Trust in Science | 1.42 (1.28-1.58) | 1.38 (1.23-1.54) | 1.39 (1.24-1.55) |
|  | 18 to 24 | 1352 (1887) | Trust in Science | 1.58 (1.35-1.86) | 1.45 (1.23-1.72) | 1.48 (1.25-1.75) |
| **Education** | College Degree | 2706 (1715) | Trust in Science | 1.00 (Reference) | 1.00 (Reference) | 1.00 (Reference) |
|  | Graduate Degree | 855 (930) | Trust in Science | 1.17 (0.98-1.39) | 1.2 (0.99-1.44) | 1.26 (1.05-1.52) |
|  | Some College | 2501 (3208) | Trust in Science | 1.13 (1-1.28) | 1.19 (1.05-1.36) | 1.17 (1.03-1.34) |
|  | High School Graduate | 3113 (3480) | Trust in Science | 1.56 (1.38-1.77) | 1.62 (1.42-1.85) | 1.57 (1.37-1.79) |
|  | Some High School or Less | 534 (600) | Trust in Science | 1.32 (1.05-1.67) | 1.5 (1.17-1.93) | 1.44 (1.12-1.85) |
| **Income** | 100K+ | 1449 (1445) | Trust in Science | 1.00 (Reference) | 1.00 (Reference) | 1.00 (Reference) |
|  | 75-100K | 970 (1028) | Trust in Science | 1.2 (0.98-1.47) | 1.12 (0.91-1.39) | 1.1 (0.89-1.36) |
|  | 50-75K | 1733 (1756) | Trust in Science | 1.04 (0.88-1.23) | 1.01 (0.84-1.21) | 0.99 (0.83-1.19) |
|  | 25-50K | 2668 (2733) | Trust in Science | 1.04 (0.89-1.21) | 1.07 (0.9-1.27) | 1.04 (0.87-1.23) |
|  | Under 25K | 2888 (2970) | Trust in Science | 0.98 (0.84-1.13) | 1.05 (0.88-1.26) | 1.02 (0.85-1.22) |
| **Insurance** | Private/Other | 4008 (4121) | Trust in Science | 1.00 (Reference) | 1.00 (Reference) | 1.00 (Reference) |
|  | Medicare/Medicaid | 5701 (5813) | Trust in Science | 0.69 (0.62-0.76) | 0.64 (0.57-0.71) | 0.64 (0.57-0.71) |

# **Supplementary Table 3:** Association between sociodemographic characteristics and not being on antidepressants, before and after adjustment for trust and emotional support

*†Model 1: Adjusted for all sociodemographic characteristics (race, ethnicity, nativity, gender, age, education, income, insurance)*

*‡Model 2: Model 1 + specified trust/social support variable*

| **Category** | **Subgroup** | **N (Weighted)** | **Added Variable** | **Unadjusted** | **†Model 1** | **‡Model 2** |
| --- | --- | --- | --- | --- | --- | --- |
| **Race** | White | 6877 (6994) | Trust in Doctors | 1.00 (Reference) | 1.00 (Reference) | 1.00 (Reference) |
|  | Black | 1444 (1299) | Trust in Doctors | 2.38 (1.99-2.84) | 2.26 (1.88-2.73) | 2.21 (1.84-2.66) |
|  | Asian American | 326 (498) | Trust in Doctors | 2.68 (1.86-3.85) | 2.53 (1.75-3.66) | 2.51 (1.73-3.62) |
|  | Native American/Pacific Islander | 222 (200) | Trust in Doctors | 1.49 (1.03-2.16) | 1.19 (0.81-1.76) | 1.16 (0.79-1.71) |
|  | Multiracial/Other | 831 (932) | Trust in Doctors | 1.84 (1.51-2.26) | 1.4 (1.12-1.74) | 1.37 (1.1-1.72) |
| **Ethnicity** | Non-Hispanic | 8383 (8219) | Trust in Doctors | 1.00 (Reference) | 1.00 (Reference) | 1.00 (Reference) |
|  | Hispanic | 1317 (1705) | Trust in Doctors | 1.89 (1.59-2.25) | 1.65 (1.36-2) | 1.67 (1.38-2.03) |
| **Ethnicity** | Non-MENA | 9315 (9523) | Trust in Doctors | 1.00 (Reference) | 1.00 (Reference) | 1.00 (Reference) |
|  | Middle Eastern or North African | 385 (400) | Trust in Doctors | 1.31 (0.99-1.75) | 0.96 (0.71-1.29) | 0.99 (0.73-1.33) |
| **Nativity** | US-born | 9301 (9450) | Trust in Doctors | 1.00 (Reference) | 1.00 (Reference) | 1.00 (Reference) |
|  | Foreign-born | 399 (473) | Trust in Doctors | 1.87 (1.38-2.53) | 1.39 (1.02-1.9) | 1.38 (1.01-1.89) |
| **Gender** | Female | 5654 (5397) | Trust in Doctors | 1.00 (Reference) | 1.00 (Reference) | 1.00 (Reference) |
|  | Male | 3906 (4361) | Trust in Doctors | 1.88 (1.68-2.1) | 1.88 (1.68-2.11) | 1.93 (1.72-2.17) |
|  | TGNB | 138 (162) | Trust in Doctors | 0.55 (0.37-0.83) | 0.43 (0.28-0.66) | 0.44 (0.29-0.66) |
| **Age Group** | 45 to 64 | 2866 (2869) | Trust in Doctors | 1.00 (Reference) | 1.00 (Reference) | 1.00 (Reference) |
|  | 65 and over | 761 (795) | Trust in Doctors | 1.05 (0.86-1.29) | 1.39 (1.12-1.72) | 1.45 (1.17-1.8) |
|  | 25 to 44 | 4719 (4369) | Trust in Doctors | 1.57 (1.4-1.76) | 1.5 (1.33-1.69) | 1.5 (1.33-1.69) |
|  | 18 to 24 | 1354 (1890) | Trust in Doctors | 1.7 (1.43-2.03) | 1.51 (1.26-1.82) | 1.54 (1.28-1.86) |
| **Education** | College Degree | 2706 (1717) | Trust in Doctors | 1.00 (Reference) | 1.00 (Reference) | 1.00 (Reference) |
|  | Graduate Degree | 857 (933) | Trust in Doctors | 1.2 (0.99-1.44) | 1.17 (0.96-1.43) | 1.24 (1.02-1.52) |
|  | Some College | 2498 (3204) | Trust in Doctors | 1.14 (1-1.29) | 1.2 (1.05-1.38) | 1.18 (1.03-1.35) |
|  | High School Graduate | 3106 (3471) | Trust in Doctors | 1.59 (1.39-1.81) | 1.62 (1.41-1.87) | 1.6 (1.39-1.85) |
|  | Some High School or Less | 533 (599) | Trust in Doctors | 1.78 (1.38-2.3) | 1.92 (1.46-2.54) | 1.86 (1.4-2.46) |
| **Income** | 100K+ | 1452 (1448) | Trust in Doctors | 1.00 (Reference) | 1.00 (Reference) | 1.00 (Reference) |
|  | 75-100K | 970 (1028) | Trust in Doctors | 1.2 (0.97-1.48) | 1.12 (0.89-1.4) | 1.09 (0.87-1.37) |
|  | 50-75K | 1726 (1749) | Trust in Doctors | 1.01 (0.85-1.21) | 0.99 (0.82-1.2) | 0.95 (0.78-1.16) |
|  | 25-50K | 2666 (2732) | Trust in Doctors | 0.93 (0.79-1.09) | 0.95 (0.79-1.14) | 0.92 (0.76-1.1) |
|  | Under 25K | 2885 (2965) | Trust in Doctors | 1.01 (0.86-1.19) | 1.06 (0.87-1.28) | 1 (0.82-1.22) |
| **Insurance** | Private/Other | 4000 (4113) | Trust in Doctors | 1.00 (Reference) | 1.00 (Reference) | 1.00 (Reference) |
|  | Medicare/Medicaid | 5700 (5810) | Trust in Doctors | 0.75 (0.68-0.84) | 0.72 (0.64-0.8) | 0.73 (0.65-0.82) |
| **Race** | White | 6890 (7009) | Trust in Pharmaceutical Companies | 1.00 (Reference) | 1.00 (Reference) | 1.00 (Reference) |
|  | Black | 1446 (1303) | Trust in Pharmaceutical Companies | 2.41 (2.01-2.88) | 2.3 (1.91-2.76) | 2.31 (1.92-2.78) |
|  | Asian American | 326 (498) | Trust in Pharmaceutical Companies | 2.68 (1.86-3.86) | 2.54 (1.75-3.67) | 2.58 (1.78-3.73) |
|  | Native American/Pacific Islander | 223 (201) | Trust in Pharmaceutical Companies | 1.51 (1.04-2.18) | 1.2 (0.82-1.77) | 1.2 (0.82-1.77) |
|  | Multiracial/Other | 834 (935) | Trust in Pharmaceutical Companies | 1.85 (1.51-2.27) | 1.4 (1.12-1.75) | 1.39 (1.11-1.73) |
| **Ethnicity** | Non-Hispanic | 8400 (8238) | Trust in Pharmaceutical Companies | 1.00 (Reference) | 1.00 (Reference) | 1.00 (Reference) |
|  | Hispanic | 1319 (1708) | Trust in Pharmaceutical Companies | 1.9 (1.59-2.26) | 1.65 (1.36-2.01) | 1.67 (1.38-2.03) |
| **Ethnicity** | Non-MENA | 9334 (9546) | Trust in Pharmaceutical Companies | 1.00 (Reference) | 1.00 (Reference) | 1.00 (Reference) |
|  | Middle Eastern or North African | 385 (400) | Trust in Pharmaceutical Companies | 1.32 (0.99-1.75) | 0.96 (0.71-1.29) | 0.97 (0.72-1.3) |
| **Nativity** | US-born | 9320 (9473) | Trust in Pharmaceutical Companies | 1.00 (Reference) | 1.00 (Reference) | 1.00 (Reference) |
|  | Foreign-born | 399 (473) | Trust in Pharmaceutical Companies | 1.87 (1.38-2.54) | 1.39 (1.02-1.9) | 1.39 (1.02-1.9) |
| **Gender** | Female | 5668 (5414) | Trust in Pharmaceutical Companies | 1.00 (Reference) | 1.00 (Reference) | 1.00 (Reference) |
|  | Male | 3911 (4367) | Trust in Pharmaceutical Companies | 1.88 (1.68-2.1) | 1.88 (1.68-2.11) | 1.88 (1.67-2.11) |
|  | TGNB | 138 (162) | Trust in Pharmaceutical Companies | 0.55 (0.37-0.83) | 0.43 (0.28-0.67) | 0.43 (0.28-0.66) |
| **Age Group** | 45 to 64 | 2873 (2876) | Trust in Pharmaceutical Companies | 1.00 (Reference) | 1.00 (Reference) | 1.00 (Reference) |
|  | 65 and over | 766 (803) | Trust in Pharmaceutical Companies | 1.04 (0.85-1.27) | 1.38 (1.12-1.71) | 1.39 (1.13-1.72) |
|  | 25 to 44 | 4724 (4376) | Trust in Pharmaceutical Companies | 1.56 (1.39-1.75) | 1.49 (1.33-1.68) | 1.49 (1.32-1.68) |
|  | 18 to 24 | 1356 (1892) | Trust in Pharmaceutical Companies | 1.7 (1.43-2.03) | 1.51 (1.26-1.82) | 1.52 (1.27-1.83) |
| **Education** | College Degree | 2705 (1716) | Trust in Pharmaceutical Companies | 1.00 (Reference) | 1.00 (Reference) | 1.00 (Reference) |
|  | Graduate Degree | 858 (934) | Trust in Pharmaceutical Companies | 1.2 (0.99-1.44) | 1.17 (0.95-1.43) | 1.18 (0.97-1.45) |
|  | Some College | 2505 (3211) | Trust in Pharmaceutical Companies | 1.13 (0.99-1.29) | 1.2 (1.04-1.37) | 1.19 (1.04-1.37) |
|  | High School Graduate | 3116 (3485) | Trust in Pharmaceutical Companies | 1.6 (1.4-1.82) | 1.63 (1.41-1.88) | 1.63 (1.42-1.88) |
|  | Some High School or Less | 535 (601) | Trust in Pharmaceutical Companies | 1.79 (1.38-2.32) | 1.94 (1.47-2.56) | 1.94 (1.47-2.57) |
| **Income** | 100K+ | 1452 (1450) | Trust in Pharmaceutical Companies | 1.00 (Reference) | 1.00 (Reference) | 1.00 (Reference) |
|  | 75-100K | 971 (1029) | Trust in Pharmaceutical Companies | 1.19 (0.96-1.48) | 1.11 (0.89-1.39) | 1.11 (0.88-1.39) |
|  | 50-75K | 1733 (1756) | Trust in Pharmaceutical Companies | 1 (0.84-1.19) | 0.98 (0.81-1.18) | 0.97 (0.8-1.17) |
|  | 25-50K | 2671 (2737) | Trust in Pharmaceutical Companies | 0.92 (0.78-1.08) | 0.94 (0.78-1.13) | 0.93 (0.77-1.12) |
|  | Under 25K | 2891 (2973) | Trust in Pharmaceutical Companies | 1.01 (0.86-1.18) | 1.05 (0.87-1.28) | 1.04 (0.86-1.27) |
| **Insurance** | Private/Other | 4007 (4121) | Trust in Pharmaceutical Companies | 1.00 (Reference) | 1.00 (Reference) | 1.00 (Reference) |
|  | Medicare/Medicaid | 5712 (5825) | Trust in Pharmaceutical Companies | 0.75 (0.68-0.84) | 0.71 (0.64-0.8) | 0.72 (0.64-0.81) |
| **Race** | White | 6858 (6972) | Social Support | 1.00 (Reference) | 1.00 (Reference) | 1.00 (Reference) |
|  | Black | 1436 (1296) | Social Support | 2.41 (2.01-2.88) | 2.3 (1.91-2.77) | 2.24 (1.86-2.7) |
|  | Asian American | 317 (488) | Social Support | 2.61 (1.81-3.76) | 2.47 (1.71-3.58) | 2.47 (1.7-3.58) |
|  | Native American/Pacific Islander | 223 (201) | Social Support | 1.5 (1.04-2.17) | 1.2 (0.81-1.77) | 1.17 (0.79-1.73) |
|  | Multiracial/Other | 829 (931) | Social Support | 1.85 (1.51-2.27) | 1.4 (1.12-1.74) | 1.38 (1.11-1.73) |
| **Ethnicity** | Non-Hispanic | 8353 (8191) | Social Support | 1.00 (Reference) | 1.00 (Reference) | 1.00 (Reference) |
|  | Hispanic | 1310 (1696) | Social Support | 1.91 (1.6-2.28) | 1.67 (1.37-2.03) | 1.69 (1.39-2.05) |
| **Ethnicity** | Non-MENA | 9283 (9492) | Social Support | 1.00 (Reference) | 1.00 (Reference) | 1.00 (Reference) |
|  | Middle Eastern or North African | 380 (395) | Social Support | 1.31 (0.98-1.75) | 0.95 (0.71-1.29) | 0.97 (0.72-1.31) |
| **Nativity** | US-born | 9271 (9422) | Social Support | 1.00 (Reference) | 1.00 (Reference) | 1.00 (Reference) |
|  | Foreign-born | 392 (465) | Social Support | 1.86 (1.36-2.52) | 1.39 (1.02-1.91) | 1.38 (1.01-1.9) |
| **Gender** | Female | 5640 (5388) | Social Support | 1.00 (Reference) | 1.00 (Reference) | 1.00 (Reference) |
|  | Male | 3883 (4334) | Social Support | 1.87 (1.67-2.09) | 1.87 (1.67-2.1) | 1.84 (1.63-2.07) |
|  | TGNB | 138 (162) | Social Support | 0.55 (0.37-0.83) | 0.43 (0.28-0.66) | 0.43 (0.28-0.65) |
| **Age Group** | 45 to 64 | 2861 (2862) | Social Support | 1.00 (Reference) | 1.00 (Reference) | 1.00 (Reference) |
|  | 65 and over | 763 (801) | Social Support | 1.05 (0.86-1.29) | 1.4 (1.13-1.73) | 1.39 (1.12-1.72) |
|  | 25 to 44 | 4698 (4351) | Social Support | 1.56 (1.39-1.75) | 1.49 (1.32-1.68) | 1.51 (1.34-1.71) |
|  | 18 to 24 | 1341 (1873) | Social Support | 1.69 (1.42-2.02) | 1.51 (1.26-1.81) | 1.54 (1.28-1.85) |
| **Education** | College Degree | 2688 (1706) | Social Support | 1.00 (Reference) | 1.00 (Reference) | 1.00 (Reference) |
|  | Graduate Degree | 853 (927) | Social Support | 1.21 (1-1.46) | 1.17 (0.96-1.44) | 1.18 (0.96-1.44) |
|  | Some College | 2500 (3201) | Social Support | 1.13 (0.99-1.28) | 1.19 (1.04-1.36) | 1.18 (1.03-1.35) |
|  | High School Graduate | 3091 (3456) | Social Support | 1.6 (1.4-1.82) | 1.63 (1.41-1.88) | 1.61 (1.39-1.85) |
|  | Some High School or Less | 531 (596) | Social Support | 1.79 (1.38-2.32) | 1.94 (1.47-2.57) | 1.91 (1.44-2.53) |
| **Income** | 100K+ | 1447 (1446) | Social Support | 1.00 (Reference) | 1.00 (Reference) | 1.00 (Reference) |
|  | 75-100K | 961 (1019) | Social Support | 1.19 (0.96-1.47) | 1.11 (0.89-1.39) | 1.09 (0.87-1.36) |
|  | 50-75K | 1724 (1747) | Social Support | 1 (0.84-1.19) | 0.98 (0.81-1.19) | 0.96 (0.79-1.16) |
|  | 25-50K | 2652 (2718) | Social Support | 0.92 (0.78-1.08) | 0.94 (0.78-1.13) | 0.91 (0.76-1.1) |
|  | Under 25K | 2878 (2958) | Social Support | 1 (0.85-1.17) | 1.04 (0.86-1.26) | 0.99 (0.82-1.21) |
| **Insurance** | Private/Other | 3987 (4101) | Social Support | 1.00 (Reference) | 1.00 (Reference) | 1.00 (Reference) |
|  | Medicare/Medicaid | 5676 (5787) | Social Support | 0.76 (0.68-0.84) | 0.72 (0.64-0.81) | 0.72 (0.64-0.81) |
| **Race** | White | 6885 (7005) | Trust in CDC | 1.00 (Reference) | 1.00 (Reference) | 1.00 (Reference) |
|  | Black | 1446 (1302) | Trust in CDC | 2.39 (2-2.86) | 2.28 (1.89-2.74) | 2.27 (1.89-2.73) |
|  | Asian American | 325 (498) | Trust in CDC | 2.71 (1.88-3.91) | 2.56 (1.76-3.71) | 2.58 (1.78-3.74) |
|  | Native American/Pacific Islander | 222 (200) | Trust in CDC | 1.51 (1.04-2.18) | 1.2 (0.82-1.77) | 1.18 (0.8-1.74) |
|  | Multiracial/Other | 834 (936) | Trust in CDC | 1.86 (1.52-2.27) | 1.4 (1.12-1.75) | 1.37 (1.1-1.71) |
| **Ethnicity** | Non-Hispanic | 8393 (8233) | Trust in CDC | 1.00 (Reference) | 1.00 (Reference) | 1.00 (Reference) |
|  | Hispanic | 1319 (1708) | Trust in CDC | 1.9 (1.6-2.26) | 1.66 (1.36-2.01) | 1.68 (1.38-2.04) |
| **Ethnicity** | Non-MENA | 9329 (9542) | Trust in CDC | 1.00 (Reference) | 1.00 (Reference) | 1.00 (Reference) |
|  | Middle Eastern or North African | 383 (399) | Trust in CDC | 1.32 (0.99-1.77) | 0.97 (0.72-1.3) | 0.97 (0.72-1.3) |
| **Nativity** | US-born | 9314 (9469) | Trust in CDC | 1.00 (Reference) | 1.00 (Reference) | 1.00 (Reference) |
|  | Foreign-born | 398 (472) | Trust in CDC | 1.89 (1.39-2.56) | 1.4 (1.02-1.91) | 1.4 (1.02-1.91) |
| **Gender** | Female | 5669 (5418) | Trust in CDC | 1.00 (Reference) | 1.00 (Reference) | 1.00 (Reference) |
|  | Male | 3903 (4358) | Trust in CDC | 1.88 (1.68-2.1) | 1.88 (1.67-2.11) | 1.9 (1.69-2.13) |
|  | TGNB | 138 (162) | Trust in CDC | 0.56 (0.37-0.83) | 0.43 (0.28-0.67) | 0.45 (0.29-0.69) |
| **Age Group** | 45 to 64 | 2874 (2876) | Trust in CDC | 1.00 (Reference) | 1.00 (Reference) | 1.00 (Reference) |
|  | 65 and over | 764 (803) | Trust in CDC | 1.04 (0.85-1.28) | 1.39 (1.12-1.71) | 1.39 (1.13-1.72) |
|  | 25 to 44 | 4721 (4373) | Trust in CDC | 1.56 (1.39-1.75) | 1.49 (1.33-1.68) | 1.48 (1.32-1.67) |
|  | 18 to 24 | 1353 (1889) | Trust in CDC | 1.71 (1.43-2.03) | 1.51 (1.26-1.82) | 1.53 (1.27-1.84) |
| **Education** | College Degree | 2704 (1715) | Trust in CDC | 1.00 (Reference) | 1.00 (Reference) | 1.00 (Reference) |
|  | Graduate Degree | 857 (932) | Trust in CDC | 1.19 (0.99-1.44) | 1.16 (0.95-1.42) | 1.2 (0.98-1.47) |
|  | Some College | 2505 (3212) | Trust in CDC | 1.13 (0.99-1.29) | 1.2 (1.04-1.37) | 1.18 (1.03-1.36) |
|  | High School Graduate | 3115 (3485) | Trust in CDC | 1.6 (1.4-1.82) | 1.63 (1.41-1.88) | 1.61 (1.4-1.86) |
|  | Some High School or Less | 531 (598) | Trust in CDC | 1.78 (1.38-2.3) | 1.93 (1.46-2.55) | 1.91 (1.45-2.53) |
| **Income** | 100K+ | 1455 (1453) | Trust in CDC | 1.00 (Reference) | 1.00 (Reference) | 1.00 (Reference) |
|  | 75-100K | 968 (1026) | Trust in CDC | 1.2 (0.96-1.48) | 1.11 (0.89-1.39) | 1.1 (0.88-1.38) |
|  | 50-75K | 1730 (1754) | Trust in CDC | 1 (0.84-1.19) | 0.98 (0.81-1.18) | 0.96 (0.79-1.16) |
|  | 25-50K | 2671 (2737) | Trust in CDC | 0.92 (0.79-1.09) | 0.94 (0.78-1.13) | 0.93 (0.77-1.11) |
|  | Under 25K | 2887 (2970) | Trust in CDC | 1.01 (0.86-1.18) | 1.05 (0.86-1.27) | 1.03 (0.85-1.25) |
| **Insurance** | Private/Other | 4006 (4118) | Trust in CDC | 1.00 (Reference) | 1.00 (Reference) | 1.00 (Reference) |
|  | Medicare/Medicaid | 5706 (5823) | Trust in CDC | 0.75 (0.68-0.84) | 0.72 (0.64-0.8) | 0.72 (0.64-0.81) |
| **Race** | White | 6882 (6999) | Trust in Science | 1.00 (Reference) | 1.00 (Reference) | 1.00 (Reference) |
|  | Black | 1445 (1302) | Trust in Science | 2.39 (2-2.86) | 2.28 (1.89-2.74) | 2.21 (1.84-2.66) |
|  | Asian American | 325 (496) | Trust in Science | 2.66 (1.85-3.84) | 2.52 (1.74-3.65) | 2.55 (1.76-3.69) |
|  | Native American/Pacific Islander | 223 (201) | Trust in Science | 1.5 (1.04-2.18) | 1.2 (0.81-1.77) | 1.15 (0.78-1.69) |
|  | Multiracial/Other | 834 (935) | Trust in Science | 1.85 (1.51-2.26) | 1.39 (1.12-1.74) | 1.38 (1.11-1.72) |
| **Ethnicity** | Non-Hispanic | 8394 (8229) | Trust in Science | 1.00 (Reference) | 1.00 (Reference) | 1.00 (Reference) |
|  | Hispanic | 1315 (1704) | Trust in Science | 1.91 (1.6-2.27) | 1.67 (1.37-2.03) | 1.65 (1.35-2) |
| **Ethnicity** | Non-MENA | 9325 (9534) | Trust in Science | 1.00 (Reference) | 1.00 (Reference) | 1.00 (Reference) |
|  | Middle Eastern or North African | 384 (399) | Trust in Science | 1.31 (0.98-1.74) | 0.95 (0.71-1.28) | 0.97 (0.72-1.3) |
| **Nativity** | US-born | 9310 (9460) | Trust in Science | 1.00 (Reference) | 1.00 (Reference) | 1.00 (Reference) |
|  | Foreign-born | 399 (473) | Trust in Science | 1.87 (1.38-2.54) | 1.39 (1.02-1.91) | 1.41 (1.03-1.92) |
| **Gender** | Female | 5662 (5409) | Trust in Science | 1.00 (Reference) | 1.00 (Reference) | 1.00 (Reference) |
|  | Male | 3908 (4359) | Trust in Science | 1.88 (1.68-2.1) | 1.89 (1.68-2.12) | 1.9 (1.7-2.14) |
|  | TGNB | 137 (161) | Trust in Science | 0.56 (0.37-0.84) | 0.44 (0.28-0.67) | 0.47 (0.31-0.72) |
| **Age Group** | 45 to 64 | 2874 (2877) | Trust in Science | 1.00 (Reference) | 1.00 (Reference) | 1.00 (Reference) |
|  | 65 and over | 764 (800) | Trust in Science | 1.05 (0.86-1.29) | 1.4 (1.13-1.73) | 1.44 (1.16-1.78) |
|  | 25 to 44 | 4719 (4369) | Trust in Science | 1.57 (1.4-1.76) | 1.5 (1.33-1.69) | 1.52 (1.35-1.71) |
|  | 18 to 24 | 1352 (1887) | Trust in Science | 1.71 (1.44-2.04) | 1.52 (1.26-1.82) | 1.55 (1.29-1.86) |
| **Education** | College Degree | 2706 (1715) | Trust in Science | 1.00 (Reference) | 1.00 (Reference) | 1.00 (Reference) |
|  | Graduate Degree | 855 (930) | Trust in Science | 1.19 (0.99-1.44) | 1.17 (0.96-1.43) | 1.23 (1.01-1.51) |
|  | Some College | 2501 (3208) | Trust in Science | 1.13 (0.99-1.29) | 1.19 (1.04-1.37) | 1.18 (1.02-1.35) |
|  | High School Graduate | 3113 (3480) | Trust in Science | 1.6 (1.4-1.82) | 1.63 (1.41-1.87) | 1.58 (1.37-1.82) |
|  | Some High School or Less | 534 (600) | Trust in Science | 1.79 (1.38-2.31) | 1.93 (1.46-2.55) | 1.85 (1.4-2.44) |
| **Income** | 100K+ | 1449 (1445) | Trust in Science | 1.00 (Reference) | 1.00 (Reference) | 1.00 (Reference) |
|  | 75-100K | 970 (1028) | Trust in Science | 1.2 (0.97-1.49) | 1.12 (0.9-1.4) | 1.1 (0.88-1.38) |
|  | 50-75K | 1733 (1756) | Trust in Science | 1 (0.84-1.2) | 0.98 (0.81-1.19) | 0.96 (0.79-1.16) |
|  | 25-50K | 2668 (2733) | Trust in Science | 0.93 (0.79-1.09) | 0.95 (0.79-1.14) | 0.92 (0.76-1.11) |
|  | Under 25K | 2888 (2970) | Trust in Science | 1.01 (0.86-1.19) | 1.06 (0.87-1.28) | 1.03 (0.84-1.24) |
| **Insurance** | Private/Other | 4008 (4121) | Trust in Science | 1.00 (Reference) | 1.00 (Reference) | 1.00 (Reference) |
|  | Medicare/Medicaid | 5701 (5813) | Trust in Science | 0.75 (0.68-0.84) | 0.71 (0.64-0.8) | 0.72 (0.64-0.8) |

# **Supplementary Table 4:** Association between sociodemographic characteristics and not being in therapy, before and after adjustment for trust and emotional support

*†Model 1: Adjusted for all sociodemographic characteristics (race, ethnicity, nativity, gender, age, education, income, insurance)*

*‡Model 2: Model 1 + specified trust/social support variable*

| **Category** | **Subgroup** | **N (Weighted)** | **Added Variable** | **Unadjusted** | **†Model 1** | **‡Model 2** |
| --- | --- | --- | --- | --- | --- | --- |
| **Race** | White | 6877 (6994) | Trust in Doctors | 1.00 (Reference) | 1.00 (Reference) | 1.00 (Reference) |
|  | Black | 1444 (1299) | Trust in Doctors | 1.2 (1.01-1.43) | 1.25 (1.04-1.49) | 1.21 (1.01-1.45) |
|  | Asian American | 326 (498) | Trust in Doctors | 1.18 (0.84-1.65) | 1.13 (0.79-1.63) | 1.09 (0.76-1.57) |
|  | Native American/Pacific Islander | 222 (200) | Trust in Doctors | 0.83 (0.55-1.26) | 0.81 (0.53-1.26) | 0.78 (0.51-1.2) |
|  | Multiracial/Other | 831 (932) | Trust in Doctors | 1.02 (0.82-1.27) | 0.98 (0.77-1.25) | 0.96 (0.75-1.23) |
| **Ethnicity** | Non-Hispanic | 8383 (8219) | Trust in Doctors | 1.00 (Reference) | 1.00 (Reference) | 1.00 (Reference) |
|  | Hispanic | 1317 (1705) | Trust in Doctors | 1.09 (0.91-1.31) | 1.09 (0.9-1.32) | 1.11 (0.91-1.35) |
| **Ethnicity** | Non-MENA | 9315 (9523) | Trust in Doctors | 1.00 (Reference) | 1.00 (Reference) | 1.00 (Reference) |
|  | Middle Eastern or North African | 385 (400) | Trust in Doctors | 0.64 (0.48-0.85) | 0.64 (0.48-0.86) | 0.66 (0.49-0.88) |
| **Nativity** | US-born | 9301 (9450) | Trust in Doctors | 1.00 (Reference) | 1.00 (Reference) | 1.00 (Reference) |
|  | Foreign-born | 399 (473) | Trust in Doctors | 1.54 (1.1-2.14) | 1.51 (1.07-2.14) | 1.52 (1.07-2.17) |
| **Gender** | Female | 5654 (5397) | Trust in Doctors | 1.00 (Reference) | 1.00 (Reference) | 1.00 (Reference) |
|  | Male | 3906 (4361) | Trust in Doctors | 1.24 (1.1-1.4) | 1.28 (1.13-1.46) | 1.35 (1.18-1.53) |
|  | TGNB | 138 (162) | Trust in Doctors | 0.35 (0.23-0.52) | 0.31 (0.2-0.47) | 0.31 (0.21-0.47) |
| **Age Group** | 45 to 64 | 2866 (2869) | Trust in Doctors | 1.00 (Reference) | 1.00 (Reference) | 1.00 (Reference) |
|  | 65 and over | 761 (795) | Trust in Doctors | 1.93 (1.46-2.55) | 2.4 (1.81-3.18) | 2.56 (1.92-3.41) |
|  | 25 to 44 | 4719 (4369) | Trust in Doctors | 1.07 (0.94-1.22) | 1.1 (0.96-1.26) | 1.11 (0.96-1.27) |
|  | 18 to 24 | 1354 (1890) | Trust in Doctors | 1.28 (1.05-1.56) | 1.27 (1.03-1.57) | 1.31 (1.06-1.61) |
| **Education** | College Degree | 2706 (1717) | Trust in Doctors | 1.00 (Reference) | 1.00 (Reference) | 1.00 (Reference) |
|  | Graduate Degree | 857 (933) | Trust in Doctors | 0.91 (0.75-1.11) | 1.01 (0.82-1.26) | 1.11 (0.89-1.38) |
|  | Some College | 2498 (3204) | Trust in Doctors | 1.15 (0.99-1.34) | 1.2 (1.02-1.4) | 1.17 (0.99-1.37) |
|  | High School Graduate | 3106 (3471) | Trust in Doctors | 1.51 (1.3-1.75) | 1.58 (1.34-1.86) | 1.56 (1.32-1.84) |
|  | Some High School or Less | 533 (599) | Trust in Doctors | 1 (0.76-1.32) | 1.17 (0.88-1.57) | 1.11 (0.83-1.49) |
| **Income** | 100K+ | 1452 (1448) | Trust in Doctors | 1.00 (Reference) | 1.00 (Reference) | 1.00 (Reference) |
|  | 75-100K | 970 (1028) | Trust in Doctors | 1.37 (1.08-1.74) | 1.26 (0.98-1.61) | 1.22 (0.95-1.56) |
|  | 50-75K | 1726 (1749) | Trust in Doctors | 1.39 (1.14-1.7) | 1.27 (1.02-1.58) | 1.19 (0.96-1.49) |
|  | 25-50K | 2666 (2732) | Trust in Doctors | 1.4 (1.17-1.68) | 1.33 (1.08-1.63) | 1.26 (1.02-1.55) |
|  | Under 25K | 2885 (2965) | Trust in Doctors | 1.14 (0.96-1.36) | 1.17 (0.95-1.45) | 1.08 (0.87-1.34) |
| **Insurance** | Private/Other | 4000 (4113) | Trust in Doctors | 1.00 (Reference) | 1.00 (Reference) | 1.00 (Reference) |
|  | Medicare/Medicaid | 5700 (5810) | Trust in Doctors | 0.62 (0.55-0.7) | 0.55 (0.49-0.63) | 0.57 (0.5-0.65) |
| **Race** | White | 6890 (7009) | Trust in Pharmaceutical Companies | 1.00 (Reference) | 1.00 (Reference) | 1.00 (Reference) |
|  | Black | 1446 (1303) | Trust in Pharmaceutical Companies | 1.22 (1.02-1.45) | 1.26 (1.05-1.51) | 1.28 (1.07-1.54) |
|  | Asian American | 326 (498) | Trust in Pharmaceutical Companies | 1.18 (0.84-1.65) | 1.13 (0.79-1.63) | 1.16 (0.81-1.67) |
|  | Native American/Pacific Islander | 223 (201) | Trust in Pharmaceutical Companies | 0.84 (0.56-1.27) | 0.82 (0.53-1.26) | 0.81 (0.53-1.24) |
|  | Multiracial/Other | 834 (935) | Trust in Pharmaceutical Companies | 1.02 (0.82-1.27) | 0.98 (0.77-1.25) | 0.97 (0.76-1.23) |
| **Ethnicity** | Non-Hispanic | 8400 (8238) | Trust in Pharmaceutical Companies | 1.00 (Reference) | 1.00 (Reference) | 1.00 (Reference) |
|  | Hispanic | 1319 (1708) | Trust in Pharmaceutical Companies | 1.09 (0.91-1.31) | 1.09 (0.89-1.32) | 1.12 (0.92-1.36) |
| **Ethnicity** | Non-MENA | 9334 (9546) | Trust in Pharmaceutical Companies | 1.00 (Reference) | 1.00 (Reference) | 1.00 (Reference) |
|  | Middle Eastern or North African | 385 (400) | Trust in Pharmaceutical Companies | 0.64 (0.48-0.85) | 0.64 (0.48-0.86) | 0.66 (0.49-0.88) |
| **Nativity** | US-born | 9320 (9473) | Trust in Pharmaceutical Companies | 1.00 (Reference) | 1.00 (Reference) | 1.00 (Reference) |
|  | Foreign-born | 399 (473) | Trust in Pharmaceutical Companies | 1.53 (1.1-2.14) | 1.51 (1.07-2.13) | 1.51 (1.07-2.14) |
| **Gender** | Female | 5668 (5414) | Trust in Pharmaceutical Companies | 1.00 (Reference) | 1.00 (Reference) | 1.00 (Reference) |
|  | Male | 3911 (4367) | Trust in Pharmaceutical Companies | 1.24 (1.09-1.4) | 1.28 (1.13-1.45) | 1.29 (1.14-1.47) |
|  | TGNB | 138 (162) | Trust in Pharmaceutical Companies | 0.35 (0.23-0.52) | 0.31 (0.2-0.47) | 0.3 (0.2-0.46) |
| **Age Group** | 45 to 64 | 2873 (2876) | Trust in Pharmaceutical Companies | 1.00 (Reference) | 1.00 (Reference) | 1.00 (Reference) |
|  | 65 and over | 766 (803) | Trust in Pharmaceutical Companies | 1.87 (1.42-2.46) | 2.32 (1.76-3.07) | 2.34 (1.77-3.1) |
|  | 25 to 44 | 4724 (4376) | Trust in Pharmaceutical Companies | 1.06 (0.93-1.21) | 1.09 (0.95-1.25) | 1.09 (0.95-1.25) |
|  | 18 to 24 | 1356 (1892) | Trust in Pharmaceutical Companies | 1.27 (1.05-1.55) | 1.26 (1.02-1.55) | 1.29 (1.04-1.59) |
| **Education** | College Degree | 2705 (1716) | Trust in Pharmaceutical Companies | 1.00 (Reference) | 1.00 (Reference) | 1.00 (Reference) |
|  | Graduate Degree | 858 (934) | Trust in Pharmaceutical Companies | 0.91 (0.74-1.11) | 1.01 (0.81-1.25) | 1.05 (0.85-1.3) |
|  | Some College | 2505 (3211) | Trust in Pharmaceutical Companies | 1.15 (0.99-1.34) | 1.2 (1.03-1.41) | 1.18 (1.01-1.38) |
|  | High School Graduate | 3116 (3485) | Trust in Pharmaceutical Companies | 1.52 (1.31-1.77) | 1.59 (1.35-1.87) | 1.61 (1.37-1.9) |
|  | Some High School or Less | 535 (601) | Trust in Pharmaceutical Companies | 1.01 (0.77-1.32) | 1.18 (0.89-1.58) | 1.19 (0.89-1.6) |
| **Income** | 100K+ | 1452 (1450) | Trust in Pharmaceutical Companies | 1.00 (Reference) | 1.00 (Reference) | 1.00 (Reference) |
|  | 75-100K | 971 (1029) | Trust in Pharmaceutical Companies | 1.36 (1.07-1.73) | 1.25 (0.98-1.6) | 1.22 (0.95-1.56) |
|  | 50-75K | 1733 (1756) | Trust in Pharmaceutical Companies | 1.38 (1.13-1.69) | 1.26 (1.01-1.56) | 1.22 (0.98-1.51) |
|  | 25-50K | 2671 (2737) | Trust in Pharmaceutical Companies | 1.39 (1.16-1.67) | 1.31 (1.07-1.61) | 1.26 (1.03-1.55) |
|  | Under 25K | 2891 (2973) | Trust in Pharmaceutical Companies | 1.14 (0.96-1.36) | 1.16 (0.94-1.44) | 1.13 (0.91-1.4) |
| **Insurance** | Private/Other | 4007 (4121) | Trust in Pharmaceutical Companies | 1.00 (Reference) | 1.00 (Reference) | 1.00 (Reference) |
|  | Medicare/Medicaid | 5712 (5825) | Trust in Pharmaceutical Companies | 0.62 (0.55-0.7) | 0.55 (0.49-0.63) | 0.57 (0.5-0.65) |
| **Race** | White | 6858 (6972) | Social Support | 1.00 (Reference) | 1.00 (Reference) | 1.00 (Reference) |
|  | Black | 1436 (1296) | Social Support | 1.22 (1.02-1.45) | 1.26 (1.05-1.51) | 1.24 (1.04-1.49) |
|  | Asian American | 317 (488) | Social Support | 1.2 (0.85-1.69) | 1.15 (0.8-1.66) | 1.15 (0.79-1.66) |
|  | Native American/Pacific Islander | 223 (201) | Social Support | 0.84 (0.55-1.26) | 0.82 (0.53-1.26) | 0.8 (0.52-1.24) |
|  | Multiracial/Other | 829 (931) | Social Support | 1.02 (0.82-1.27) | 0.98 (0.77-1.25) | 0.98 (0.77-1.24) |
| **Ethnicity** | Non-Hispanic | 8353 (8191) | Social Support | 1.00 (Reference) | 1.00 (Reference) | 1.00 (Reference) |
|  | Hispanic | 1310 (1696) | Social Support | 1.09 (0.91-1.3) | 1.08 (0.89-1.32) | 1.09 (0.89-1.32) |
| **Ethnicity** | Non-MENA | 9283 (9492) | Social Support | 1.00 (Reference) | 1.00 (Reference) | 1.00 (Reference) |
|  | Middle Eastern or North African | 380 (395) | Social Support | 0.65 (0.48-0.86) | 0.64 (0.48-0.87) | 0.65 (0.48-0.88) |
| **Nativity** | US-born | 9271 (9422) | Social Support | 1.00 (Reference) | 1.00 (Reference) | 1.00 (Reference) |
|  | Foreign-born | 392 (465) | Social Support | 1.57 (1.12-2.19) | 1.54 (1.09-2.19) | 1.54 (1.08-2.19) |
| **Gender** | Female | 5640 (5388) | Social Support | 1.00 (Reference) | 1.00 (Reference) | 1.00 (Reference) |
|  | Male | 3883 (4334) | Social Support | 1.24 (1.09-1.4) | 1.28 (1.13-1.46) | 1.27 (1.11-1.44) |
|  | TGNB | 138 (162) | Social Support | 0.34 (0.23-0.52) | 0.31 (0.2-0.47) | 0.31 (0.2-0.47) |
| **Age Group** | 45 to 64 | 2861 (2862) | Social Support | 1.00 (Reference) | 1.00 (Reference) | 1.00 (Reference) |
|  | 65 and over | 763 (801) | Social Support | 1.92 (1.46-2.53) | 2.39 (1.8-3.16) | 2.38 (1.8-3.15) |
|  | 25 to 44 | 4698 (4351) | Social Support | 1.08 (0.94-1.23) | 1.1 (0.96-1.26) | 1.11 (0.97-1.27) |
|  | 18 to 24 | 1341 (1873) | Social Support | 1.29 (1.05-1.57) | 1.27 (1.03-1.57) | 1.28 (1.04-1.59) |
| **Education** | College Degree | 2688 (1706) | Social Support | 1.00 (Reference) | 1.00 (Reference) | 1.00 (Reference) |
|  | Graduate Degree | 853 (927) | Social Support | 0.93 (0.76-1.14) | 1.02 (0.82-1.27) | 1.03 (0.83-1.28) |
|  | Some College | 2500 (3201) | Social Support | 1.15 (0.99-1.34) | 1.2 (1.03-1.41) | 1.2 (1.02-1.4) |
|  | High School Graduate | 3091 (3456) | Social Support | 1.52 (1.31-1.77) | 1.6 (1.36-1.89) | 1.59 (1.35-1.88) |
|  | Some High School or Less | 531 (596) | Social Support | 1.01 (0.77-1.33) | 1.19 (0.89-1.6) | 1.18 (0.88-1.58) |
| **Income** | 100K+ | 1447 (1446) | Social Support | 1.00 (Reference) | 1.00 (Reference) | 1.00 (Reference) |
|  | 75-100K | 961 (1019) | Social Support | 1.36 (1.07-1.73) | 1.25 (0.98-1.61) | 1.24 (0.97-1.59) |
|  | 50-75K | 1724 (1747) | Social Support | 1.35 (1.1-1.66) | 1.24 (1-1.54) | 1.23 (0.99-1.53) |
|  | 25-50K | 2652 (2718) | Social Support | 1.38 (1.15-1.66) | 1.31 (1.07-1.61) | 1.3 (1.06-1.59) |
|  | Under 25K | 2878 (2958) | Social Support | 1.11 (0.93-1.33) | 1.15 (0.93-1.42) | 1.12 (0.9-1.39) |
| **Insurance** | Private/Other | 3987 (4101) | Social Support | 1.00 (Reference) | 1.00 (Reference) | 1.00 (Reference) |
|  | Medicare/Medicaid | 5676 (5787) | Social Support | 0.63 (0.55-0.71) | 0.56 (0.49-0.64) | 0.56 (0.49-0.64) |
| **Race** | White | 6885 (7005) | Trust in CDC | 1.00 (Reference) | 1.00 (Reference) | 1.00 (Reference) |
|  | Black | 1446 (1302) | Trust in CDC | 1.21 (1.02-1.44) | 1.25 (1.05-1.5) | 1.24 (1.04-1.49) |
|  | Asian American | 325 (498) | Trust in CDC | 1.18 (0.84-1.65) | 1.14 (0.79-1.63) | 1.14 (0.79-1.63) |
|  | Native American/Pacific Islander | 222 (200) | Trust in CDC | 0.85 (0.56-1.28) | 0.82 (0.53-1.27) | 0.8 (0.52-1.23) |
|  | Multiracial/Other | 834 (936) | Trust in CDC | 1.02 (0.82-1.28) | 0.98 (0.77-1.25) | 0.95 (0.75-1.21) |
| **Ethnicity** | Non-Hispanic | 8393 (8233) | Trust in CDC | 1.00 (Reference) | 1.00 (Reference) | 1.00 (Reference) |
|  | Hispanic | 1319 (1708) | Trust in CDC | 1.1 (0.92-1.31) | 1.09 (0.9-1.32) | 1.12 (0.92-1.36) |
| **Ethnicity** | Non-MENA | 9329 (9542) | Trust in CDC | 1.00 (Reference) | 1.00 (Reference) | 1.00 (Reference) |
|  | Middle Eastern or North African | 383 (399) | Trust in CDC | 0.64 (0.48-0.86) | 0.64 (0.48-0.86) | 0.64 (0.48-0.87) |
| **Nativity** | US-born | 9314 (9469) | Trust in CDC | 1.00 (Reference) | 1.00 (Reference) | 1.00 (Reference) |
|  | Foreign-born | 398 (472) | Trust in CDC | 1.53 (1.1-2.14) | 1.51 (1.06-2.13) | 1.51 (1.07-2.14) |
| **Gender** | Female | 5669 (5418) | Trust in CDC | 1.00 (Reference) | 1.00 (Reference) | 1.00 (Reference) |
|  | Male | 3903 (4358) | Trust in CDC | 1.24 (1.09-1.4) | 1.28 (1.13-1.45) | 1.3 (1.14-1.48) |
|  | TGNB | 138 (162) | Trust in CDC | 0.35 (0.23-0.52) | 0.31 (0.2-0.47) | 0.33 (0.21-0.5) |
| **Age Group** | 45 to 64 | 2874 (2876) | Trust in CDC | 1.00 (Reference) | 1.00 (Reference) | 1.00 (Reference) |
|  | 65 and over | 764 (803) | Trust in CDC | 1.88 (1.43-2.47) | 2.34 (1.77-3.09) | 2.36 (1.78-3.12) |
|  | 25 to 44 | 4721 (4373) | Trust in CDC | 1.07 (0.94-1.22) | 1.09 (0.96-1.25) | 1.08 (0.94-1.24) |
|  | 18 to 24 | 1353 (1889) | Trust in CDC | 1.28 (1.05-1.56) | 1.26 (1.02-1.56) | 1.28 (1.03-1.57) |
| **Education** | College Degree | 2704 (1715) | Trust in CDC | 1.00 (Reference) | 1.00 (Reference) | 1.00 (Reference) |
|  | Graduate Degree | 857 (932) | Trust in CDC | 0.91 (0.74-1.11) | 1.01 (0.81-1.25) | 1.05 (0.85-1.31) |
|  | Some College | 2505 (3212) | Trust in CDC | 1.15 (0.99-1.34) | 1.2 (1.03-1.41) | 1.18 (1.01-1.38) |
|  | High School Graduate | 3115 (3485) | Trust in CDC | 1.52 (1.31-1.77) | 1.59 (1.35-1.88) | 1.57 (1.33-1.85) |
|  | Some High School or Less | 531 (598) | Trust in CDC | 1 (0.77-1.32) | 1.18 (0.88-1.57) | 1.17 (0.87-1.56) |
| **Income** | 100K+ | 1455 (1453) | Trust in CDC | 1.00 (Reference) | 1.00 (Reference) | 1.00 (Reference) |
|  | 75-100K | 968 (1026) | Trust in CDC | 1.37 (1.08-1.74) | 1.25 (0.98-1.61) | 1.23 (0.96-1.58) |
|  | 50-75K | 1730 (1754) | Trust in CDC | 1.38 (1.13-1.69) | 1.26 (1.01-1.56) | 1.22 (0.98-1.52) |
|  | 25-50K | 2671 (2737) | Trust in CDC | 1.39 (1.16-1.67) | 1.32 (1.07-1.61) | 1.28 (1.04-1.57) |
|  | Under 25K | 2887 (2970) | Trust in CDC | 1.14 (0.96-1.36) | 1.17 (0.94-1.44) | 1.12 (0.91-1.39) |
| **Insurance** | Private/Other | 4006 (4118) | Trust in CDC | 1.00 (Reference) | 1.00 (Reference) | 1.00 (Reference) |
|  | Medicare/Medicaid | 5706 (5823) | Trust in CDC | 0.62 (0.55-0.7) | 0.55 (0.49-0.63) | 0.56 (0.49-0.64) |
| **Race** | White | 6882 (6999) | Trust in Science | 1.00 (Reference) | 1.00 (Reference) | 1.00 (Reference) |
|  | Black | 1445 (1302) | Trust in Science | 1.21 (1.01-1.44) | 1.24 (1.04-1.49) | 1.21 (1.01-1.44) |
|  | Asian American | 325 (496) | Trust in Science | 1.17 (0.83-1.64) | 1.12 (0.78-1.6) | 1.12 (0.78-1.6) |
|  | Native American/Pacific Islander | 223 (201) | Trust in Science | 0.84 (0.55-1.26) | 0.81 (0.53-1.25) | 0.78 (0.5-1.2) |
|  | Multiracial/Other | 834 (935) | Trust in Science | 1.02 (0.82-1.27) | 0.98 (0.77-1.24) | 0.96 (0.76-1.23) |
| **Ethnicity** | Non-Hispanic | 8394 (8229) | Trust in Science | 1.00 (Reference) | 1.00 (Reference) | 1.00 (Reference) |
|  | Hispanic | 1315 (1704) | Trust in Science | 1.1 (0.92-1.31) | 1.09 (0.9-1.33) | 1.08 (0.89-1.32) |
| **Ethnicity** | Non-MENA | 9325 (9534) | Trust in Science | 1.00 (Reference) | 1.00 (Reference) | 1.00 (Reference) |
|  | Middle Eastern or North African | 384 (399) | Trust in Science | 0.65 (0.49-0.86) | 0.64 (0.48-0.87) | 0.65 (0.49-0.88) |
| **Nativity** | US-born | 9310 (9460) | Trust in Science | 1.00 (Reference) | 1.00 (Reference) | 1.00 (Reference) |
|  | Foreign-born | 399 (473) | Trust in Science | 1.53 (1.1-2.13) | 1.51 (1.07-2.14) | 1.54 (1.09-2.18) |
| **Gender** | Female | 5662 (5409) | Trust in Science | 1.00 (Reference) | 1.00 (Reference) | 1.00 (Reference) |
|  | Male | 3908 (4359) | Trust in Science | 1.24 (1.1-1.4) | 1.28 (1.13-1.45) | 1.29 (1.14-1.47) |
|  | TGNB | 137 (161) | Trust in Science | 0.35 (0.23-0.53) | 0.31 (0.2-0.47) | 0.34 (0.22-0.52) |
| **Age Group** | 45 to 64 | 2874 (2877) | Trust in Science | 1.00 (Reference) | 1.00 (Reference) | 1.00 (Reference) |
|  | 65 and over | 764 (800) | Trust in Science | 1.87 (1.42-2.47) | 2.34 (1.77-3.09) | 2.41 (1.82-3.19) |
|  | 25 to 44 | 4719 (4369) | Trust in Science | 1.07 (0.94-1.22) | 1.1 (0.96-1.26) | 1.11 (0.97-1.27) |
|  | 18 to 24 | 1352 (1887) | Trust in Science | 1.3 (1.07-1.59) | 1.29 (1.05-1.59) | 1.31 (1.06-1.62) |
| **Education** | College Degree | 2706 (1715) | Trust in Science | 1.00 (Reference) | 1.00 (Reference) | 1.00 (Reference) |
|  | Graduate Degree | 855 (930) | Trust in Science | 0.91 (0.75-1.12) | 1.01 (0.81-1.25) | 1.07 (0.86-1.33) |
|  | Some College | 2501 (3208) | Trust in Science | 1.15 (0.99-1.33) | 1.19 (1.02-1.39) | 1.17 (1-1.37) |
|  | High School Graduate | 3113 (3480) | Trust in Science | 1.51 (1.3-1.75) | 1.58 (1.34-1.86) | 1.53 (1.3-1.8) |
|  | Some High School or Less | 534 (600) | Trust in Science | 1 (0.76-1.31) | 1.17 (0.88-1.56) | 1.11 (0.83-1.49) |
| **Income** | 100K+ | 1449 (1445) | Trust in Science | 1.00 (Reference) | 1.00 (Reference) | 1.00 (Reference) |
|  | 75-100K | 970 (1028) | Trust in Science | 1.36 (1.07-1.73) | 1.25 (0.97-1.6) | 1.22 (0.95-1.56) |
|  | 50-75K | 1733 (1756) | Trust in Science | 1.36 (1.11-1.67) | 1.25 (1-1.55) | 1.22 (0.98-1.52) |
|  | 25-50K | 2668 (2733) | Trust in Science | 1.38 (1.15-1.65) | 1.31 (1.06-1.6) | 1.27 (1.04-1.56) |
|  | Under 25K | 2888 (2970) | Trust in Science | 1.13 (0.94-1.34) | 1.16 (0.94-1.43) | 1.12 (0.91-1.39) |
| **Insurance** | Private/Other | 4008 (4121) | Trust in Science | 1.00 (Reference) | 1.00 (Reference) | 1.00 (Reference) |
|  | Medicare/Medicaid | 5701 (5813) | Trust in Science | 0.62 (0.54-0.7) | 0.55 (0.48-0.63) | 0.55 (0.48-0.63) |
